# Insights from modelling sixteen years of climatic and fumonisin patterns in maize in South Africa

**DOI:** 10.1038/s41598-024-60904-y

**Published:** 2024-05-21

**Authors:** Sefater Gbashi, Oluwasola Abayomi Adelusi, Patrick Berka Njobeh

**Affiliations:** Department of Biotechnology and Food Technology, Faculty of Science, University of Johannesburg, Doornfontein Campus, P.O Box 17011, Gauteng, 2028 South Africa

**Keywords:** Mycotoxins, Fumonisin, Climate change, Machine learning, Predictive modelling, Environmental sciences, Computational models, Data acquisition, Data mining, Data processing, Machine learning, Statistical methods

## Abstract

Mycotoxin contamination of agricultural commodities is a global public health problem that has remained elusive to various mitigation approaches, particularly in developing countries. Climate change and its impact exacerbates South Africa’s vulnerability to mycotoxin contamination, and significantly threatens its’s food systems, public health, and agro-economic development. Herein we analyse sixteen years (2005/2006–2020/2021) of annual national meteorological data on South Africa which reveals both systematic and erratic variability in critical climatic factors known to influence mycotoxin contamination in crops. Within the same study period, data on fumonisin (FB) monitoring show clear climate-dependent trends. The strongest positive warming trend is observed between 2018/2019 and 2019/2020 (0.51 °C/year), and a strong positive correlation is likewise established between FB contamination and temperature (r ranging from 0.6 to 0.9). Four machine learning models, viz support vector machines, eXtreme gradient boosting, random forest, and orthogonal partial least squares, are generalized on the historical data with suitable performance (RMSE as low as 0.00). All the adopted models are able to predict future FB contamination patterns with reasonable precision (R^2^ ranging from 0.34 to 1.00). The most important model feature for predicting average FB contamination (YA) is the historical pattern of average FB contamination in maize within the region (ΣFBs_avg). The two most significant features in modelling maximum FB contamination (YM) are minimum temperature from the CMIP6 data (Pro_tempMIN) and observed precipitation from the CRU data (O_prep). Our study provides strong evidence of the impact of climate change on FB in South Africa and reiterates the significance of machine learning modelling in predicting mycotoxin contamination in light of changing climatic conditions, which could facilitate early warnings and the adoption of relevant mitigation measures that could help in mycotoxin risk management and control.

## Introduction

Mycotoxins are a group of toxic secondary metabolites produced by some types of fungi and found in a wide range of food and other agricultural commodities. Fumonisins (FBs), a particularly toxic group of mycotoxins (with some members categorised as group 2B carcinogens) are ubiquitous and highly pervasive in South Africa, a country with favourable climatic conditions and as such, are a very important class of mycotoxins. In fact, FBs in 1988 were first isolated and characterized in South Africa in response to incessant incidenfces of oesophageal cancer in the country that was subsequently linked to this mycotoxin group^1,2^. *Fusarium* species are the primary producers of this group of poisonous fungal compounds, notably *Fusarium verticioides*, *F*. *oxysporum*, *F. proliferatum*, *F*. *nygamai*, *F*. *dlamini*, *F*. *napiforme*, and related species^1^, known to be vulnerable to changing climatic conditions.

The incidence rate of FBs in maize in South Africa and other sub-Saharan African countries often exceeds 90%^3–5^. Maize in particular, is a favoured substrate for FB contamination, however, they have also been identified in other grains such as sorghum, millet, and wheat, as well as other agricultural commodities. The *Codex Alimentarius* Commission recently set maximum levels (MLs) for FBs at 4000 μg/kg for raw maize and 2000 μg/kg for maize flour and meal^6,7^. Health legislation in South Africa have incorporated these MLs^6^, nevertheless, historical FB statistics from South Africa indicate that a significant portion of rural crops are consistently contaminated at levels above these MRLs^6,8^. In other parts of the world where oesophageal cancer is frequent in humans, such as Vietnam and China, high levels of FBs have been found in locally farmed maize. Animal diseases including Leukoencephalomalacia in horses and pulmonary oedema in pigs have also been lined to FBs^9–11^.

Studies have revealed that environmental variables like temperature, precipitation, humidity, drought, etc. affect the patterns of mycotoxin contamination. The geoclimatic conditions in which crops are grown are essential for the proliferation of fungi and subsequent development of mycotoxins^7,12–14^, because these conditions increase the likelihood of plant stress/illnesses, fungal/pest attacks on the crops, and ecological adaptation of microorganisms including mycotoxigenic fungi, possibly leading to increased virulence and associated metabolism of mycotoxins (including entirely new metabolites that are equally toxic), which have been linked to fungal defence/survival mechanisms. In light of the eminent changing climatic conditions and extreme weather events in South Africa coupled with the associated risks of mycotoxin-mediated food safety and public health issues, a critical knowledge of climate-dependent mycotoxin contamination patterns as well as the ability to pre-empt possible climate-induced mycotoxin risks is imperative for risk planning, preparedness, and adopting a prophylactic approach in minimising human and animal exposure to mycotoxins and limiting its socio-economic and health impact in the country.

Machine learning models are known to be very powerful tools having strong predictive prowess in generalizing complex nonlinear relationships in real-world data. They have been used to infer associations in measured meteorological indices from historical data and predict future occurrences of climate-dependent events^15–17^. Studies have also shown the efficacy of predictive models in forecasting risk of FB contamination in maize and small grains. For example, de la Campa et al.^18^ modelled and predicted risk of FBs in natural and genetically modified maize using climate data pertinent to the growing period from 10 days prior to 50% silking to 14 days post-silking. The authors observed that information contained in meteorological data accounted for about 47% of the statistical variance in FB contamination patterns, while insect damage and hybrid type accounted for 17 and 14% of the patterns in FB contamination, respectively. In order to forecast *Fusarium* Head Blight in wheat, Xiao et al.^19^ used two predictive models, i.e., the Relevance Vector Machine (RVM) and the Logistic model. The authors reported that RVM performed well on a small number of samples, but the universality of the model's parameters was not high, suggesting that the parameters will change with space and over time. They recommended that future studies should adopt deep learning methods and a sizable dataset as input. Using an integrated modelling approach, Liu et al.^20^ developed PREMA and PREFUM, as forecasting models, for predicting aflatoxin (AF) and FB contamination of maize grown in Serbia, respectively.

Within the African region, there is a conspicuous scarcity of such well-developed predictive models for unravelling and forecasting climate-dependent mycotoxin contamination patterns. As such, herein, we propose using four machine learning models that includes support vector machines (SVM), extreme gradient boosting regressor (XGB), random forest regressor (RF), and orthogonal partial least squares regression (OPLS) to model and predict climate-dependent FB contamination patterns in maize in South Africa based on 16 years monitoring historical data.

## Results

This study evaluated 16 consecutive years (2005/2006–2020/2021) of climatic trend and how this period can be understood to impact on FB contamination of maize in South Africa. An explorative summary of the data generated is presented in Table 1.

**Table 1:** Descriptive and exploratory analysis.

Variable	Min (n = 16)	Max (n = 16)	Mean (n = 16)	Std (n = 16)	Skewness	Kurtosis	Q1 (25)	Q2 (50)	Q3 (75)	Rate of Change
ΣFBs_avg	100.00	970.00	319.38	226.08	1.70	2.35	185.00	247.50	361.25	− 54.38
Incidence	21.71	100.00	45.70	17.72	1.67	3.75	37.81	44.14	50.83	− 4.89
ΣFBs_max	1401.00	34,740.00	7105.75	7695.33	3.14	8.87	4305.00	5365.00	5960.75	− 14.19
O_tempMEAN	17.89	19.27	18.44	0.37	0.59	− 0.21	18.19	18.42	18.61	0.01
O_tempMAX	25.35	27.17	26.14	0.48	0.46	− 0.23	25.90	26.08	26.40	0.02
O_tempMIN	10.34	11.43	10.79	0.32	0.15	− 0.73	10.58	10.86	10.95	− 0.01
O_prep	373.61	575.61	458.47	56.82	0.54	− 0.37	429.06	451.06	483.06	− 3.06
Pro_WSI	1.08	13.21	7.88	3.43	− 0.37	− 0.72	5.54	8.14	10.71	− 0.17
Pro_HUM	50.54	55.96	52.79	1.08	1.08	3.64	52.41	52.77	53.01	0.03
Pro_prep	727.72	829.61	772.16	29.57	0.10	− 0.81	750.06	773.84	792.11	2.45
Pro_tempMIN	10.81	11.51	11.09	0.18	0.62	0.09	10.96	11.08	11.20	0.02
Pro_tempMEAN	18.19	18.92	18.41	0.18	1.42	2.03	18.29	18.37	18.49	0.02
Pro_tempMAX	25.56	26.47	25.81	0.23	1.50	1.98	25.66	25.73	25.95	0.02
Pro_GSL	351.91	357.73	355.36	1.54	− 0.53	0.11	354.93	355.17	356.30	0.01
Pro_ADI	− 0.06	0.19	0.01	0.06	2.23	4.91	− 0.01	0.00	0.00	0.00

Key: *Std* standard deviation, *Q1* 1st quartile (25th percentile), *Q2* 2nd quartile (50th percentile), *Q3* 3rd quartile (75th percentile), *Rate of Change* rate of change per annum over the study period. Units: ΣFBs_avg (µg/kg), ΣFBs_max (µg/kg), Incidence (%), Pro_WSI (days), Pro_GSL (days), Pro_ADI (SPI Index), O_prep and Pro_prep (mm/year), and all temperature variables i.e., O_tempMEAN, O_tempMAX, O_tempMIN, Pro_tempMIN, Pro_tempMEAN, and Pro_tempMAX are measured in °C. Rate of Change is estimated in units of original data per year. Min, Max, Mean, Std, and percentiles are measured in the same units as the original data. Skewness and kurtosis are dimensionless.

### Climatic trends and FB contamination patterns in South Africa

**Figure 1:**
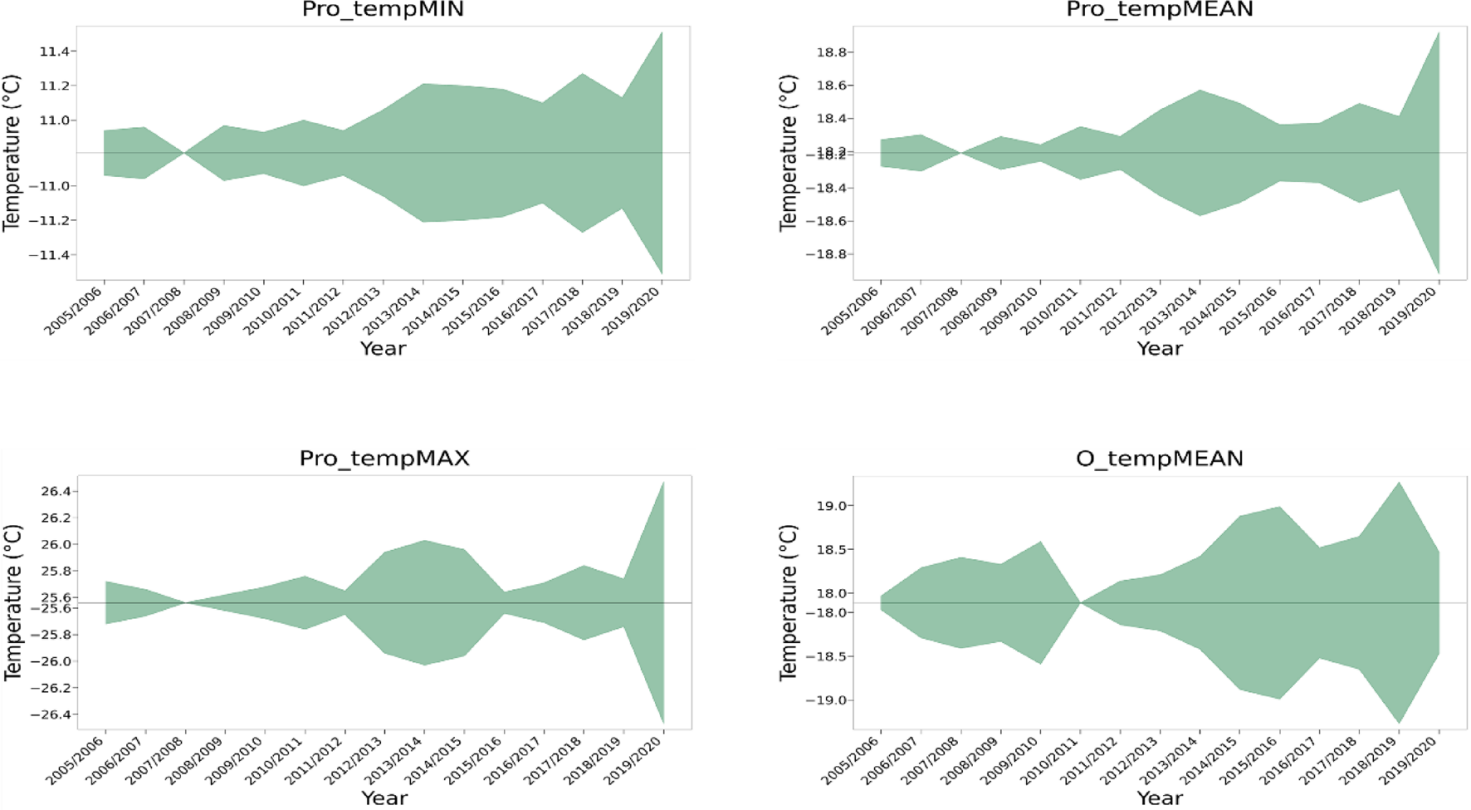
Timeseries plot showing temperature trend.

South African meteorological data within the study period demonstrate an upward trend of CMIP6 temperature data features (Pro_tempMEAN, Pro_tempMIN, and Pro_tempMAX) at an average rate of + 0.02 °C/year, which is consistent with those of other reports^21–23^. These observed temperature data structures can be clearly visualized on Fig. 1 and are supported by the ADF test of trend/stationarity, which indicates that amongst the climatological data features, Pro_tempMIN (ADF statistic = 0.262, *p* = 0.976) was the data feature with the strongest trending characteristics aside Pro_prep (Supplementary Table 1). Pro_tempMAX (ADF statistic = − 0.140, *p* = 0.945) and Pro_tempMEAN (ADF statistic = − 2.096, *p* = 0.246) likewise showed some trending characteristics. Pro_prep (ADF statistic = 0.452, *p* = 0.983) demonstrated the strongest trending features of all climatological variables.

All other climatological data features demonstrated some level of trend, albeit weak. The absolute highest (Pro_tempMAX = 26.47 °C) and lowest temperatures (Pro_tempMIN = 10.81 °C) were recorded for 2019/2020 and 2007/2008. The strongest positive warming trend for O_tempMEAN was observed between 2010/2011 and 2015/2016 (0.18 °C/year), while that for Pro_tempMean was 0.51 °C/year and occurred between 2018/2019 and 2019/2020. The CRU temperature readings (O_tempMEAN, O_tempMIN, and O_tempMAX) was noisier (more variable) than the CMIP6 temperature estimates within the study period. Though no clear time-dependent structures were observed in this study in the case of humidity, wet spell index, and precipitation (i.e., O_prep) based on the ADF analysis (*p* ≤ 0.05), O_prep showed an overall downward trajectory over the study period (− 3.06 °C/year) indicating lesser rainfall over time (Table 1). Data on maximum FB levels in maize (i.e., ΣFBs_max) showed an increasing trend (Supplementary Table 1). Of all data variables examined in this study, ΣFBs_avg showed the strongest time-dependent trend based on the ADF analysis (ADF statistic = 6.106, *p* = 1.00), followed by ΣFBs_max (ADF statistic = 0.586, *p* = 0.987), while FB incidence rate showed a weak trending pattern (ADF statistic = − 1.535, *p* = 0.516) (Supplementary Table 1). Pro_HUM demonstrated the most random and time-independent structure characteristics (ADF statistic = − 3.581, *p* = 0.006).

### Correlation and causality associations between data features

**Figure 2:**
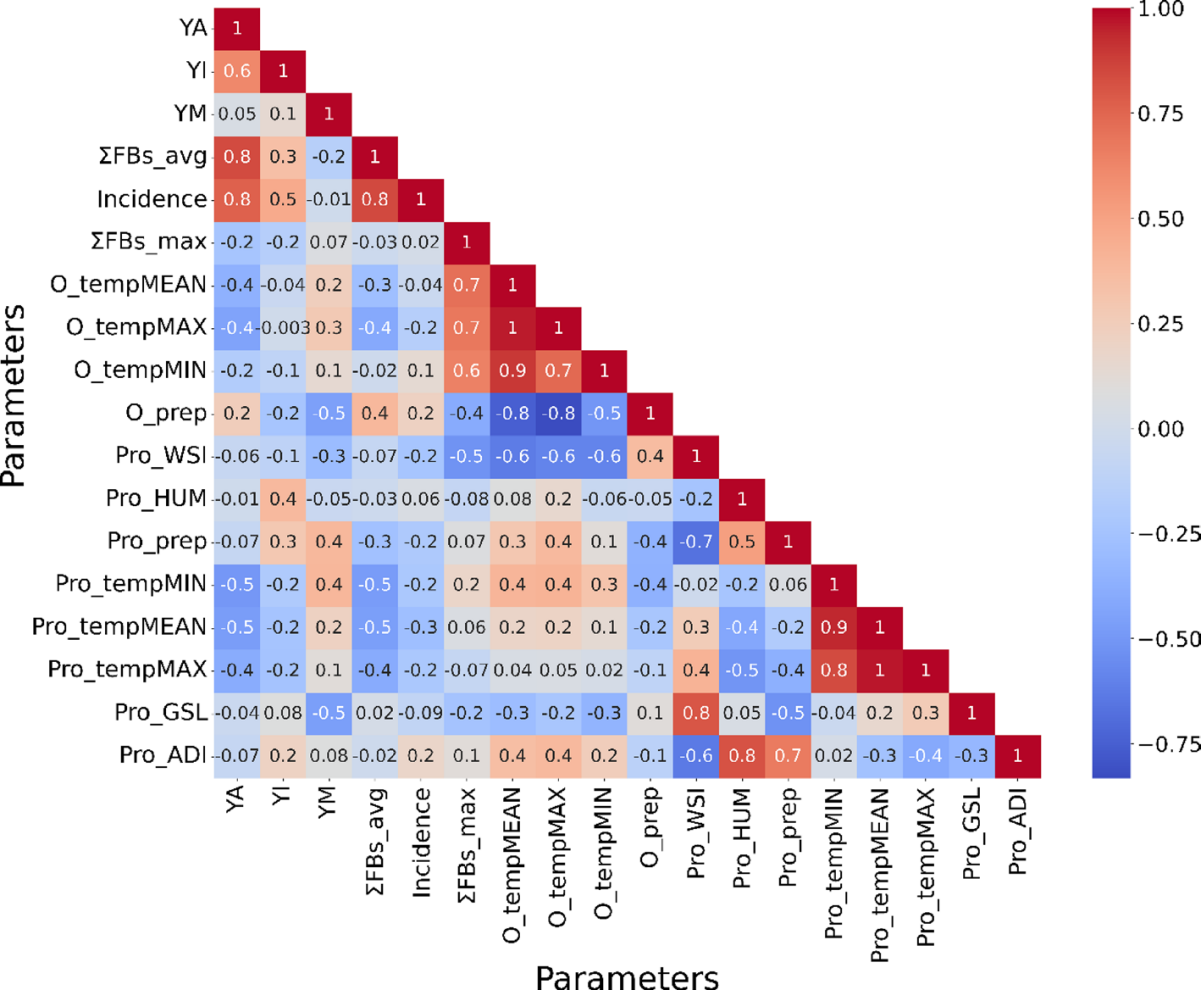
Person’s bivariate pairwise correlation between data features. The colour scale is a cool-warm gradient that conveys the strength and direction of correlations in data where deeper shades of red signify Pearson's correlation coefficients approaching 1 (suggesting positive/perfect correlation and a direct relationship) while deeper shades of blue represent Pearson's correlation values nearing -1 (indicating reverse correlation and an inverse relationship).

The Pearson’s bivariate correlation coefficients (Fig. 2) show varying degrees of linear associations between all pairwise combinations of the variables in the data. As expected, there was a robust positive linear association between FB incidence rate and average contamination levels (ΣFBs_avg) (r = 0.9). Strong positive correlations between maximum FB contamination (ΣFBs_max) and CMIP6 mean, minimum, and maximum temperatures were also observed (r = 0.9). CRU mean, maximum, and minimum temperatures (i.e., O_tempMEAN, O_tempMAX, and O_tempMIN, respectively) likewise had strong positive correlation (range: 0.6 to 0.7) with ΣFBs_max. The results of Granger causality (Supplementary Table 2) indicate that the previous two years’ length of growing season and wet spell index are useful in predicting FB incidence rates in South Africa (*p* ≤ 0.05 lag 2), while the previous two seasons’ values of wet spell index Granger causes maximum FB contamination rates (*p* ≤ 0.05 lag 2). Also, the previous season’s and the previous two season’s data on Pro_Hum (humidity), Pro_tempMEAN, and Pro_tempMAX all Granger causes maximum FB contamination rates (*p* ≤ 0.05 lag 1 and 2). Moreover, precipitation (O_prep) and length of growing season (Pro_GSL) were negatively correlated (r = − 0.5) with YM. Herein, it is observed that O_tempMEAN Granger causes O_prep at both lag 1 and 2, implying that both the previous year’ and previous 2 years’ temperature data (tempMEAN) are useful in predicting precipitation in South Africa.

### Predictive modelling using machine learning

In this study, 4 different machine learning models (SVM, XGB, RF, and OPLS) were utilized to predict FB contamination patterns in maize in South Africa. The models were trained on historical data between 2005/2006 and 2018/2019, and the generalized models used to predict FB contamination for the planting season 2019/2020. Model fitness and predictive capabilities were evaluated by examining the RMSE, MAE, and R^2^ between the predicted and experimental values. The closer the RMSE value is to zero, the more fit the model is. The results for model fit parameters and diagnostics were variable, as the performance of the various models varied based on the response variable and metric under consideration. For example, the OPLS model had the lowest prediction residual for YA response and the highest residual for YM variable. The RF model had the lowest RMSE for YA response, while the SVM model had the lowest RMSE, MAE, and R^2^ for YI and YM responses. All models fitted better to the YI (FB incidence rate) data with R^2^ ranging from 0.77 to 1.00, followed by YA (average FB contamination) data with R^2^ ranging from 0.76 to 0.95 (Table 2). Maximum FB contamination (YM) had the most uncorrelated/non-systematic variations indicating a higher level of noise and randomness in the variable.

**Table 2:** Model performance parameters.

Response variable	Model	RMSE	MAE	R^2^	Predicted	Residual
Average FBs contamination	SVM	133.46	80.88	0.81	241.84	141.84
	XGB	57.07	39.78	0.93	218.69	118.69
	RF	53.02	43.06	0.95	357.91	257.91
	OPLS	69.00	60.23	0.76	102.45	2.44
Incidence rate	SVM	0.00	0.00	1.00	43.53	21.82
	XGB	5.29	3.36	0.77	33.05	11.34
	RF	3.61	2.85	0.93	32.39	10.68
	OPLS	5.36	4.84	0.99	59.35	37.64
Max contamination	SVM	7812.45	3110.33	0.34	4271.70	− 1101.30
	XGB	3181.93	1254.05	0.96	9810.27	4437.27
	RF	2982.75	1826.02	0.91	11,994.23	6621.23
	OPLS	5425.33	3629.80	0.75	26,543.20	21,170.20

Key: *SVM* support vector machine, *XGB* extreme gradient boosting regressor, *RF* random forest regressor, *OPLS* orthogonal partial least squares, *RMSE* room mean square error, *MAE* mean average error, *R*^*2*^ coefficient of determination.

Due to the differential performances of the models and the variability of the model performance parameters, it was challenging to determine which model performed the best by mere observation of the outcomes. Consequently, the Kendall W test of concordance was used to rank the efficacy of all employed models based on model performance parameters shown in Supplementary Table 4. The results of the test indicate discrepancies in the performances and learning characteristics of the different machine learning models, albeit these differences are not statistically significant (Kendall W = 0.203, *p* = 0.063) (Supplementary Table 4). The Friedman rank score provides an insight into the overall ranking of the models (Supplementary Table 4). In this study, lower rank scores imply overall superior performances (in terms of accuracy in approximating the patterns in the data and predicting future values of the dependent variables) as compared to higher scores. For the performance ranking, RF was the most competent model with a mean rank score of 2.00, followed by XGB (2.08), SVM (2.67), and lastly OPLS (3.25). The average predicted values for YA, YI, and YM for three of the four machine learning models (RF, XGB, and SVM) for the year 2019/2020 maize growing season were 272.81 ug/kg (actual = 100 ug/kg), 36.32% (actual = 21.71%), and 8692.07 ug/kg (actual = 5373.00 ug/kg), respectively.

### Variable importance and significance

**Figure 3:**
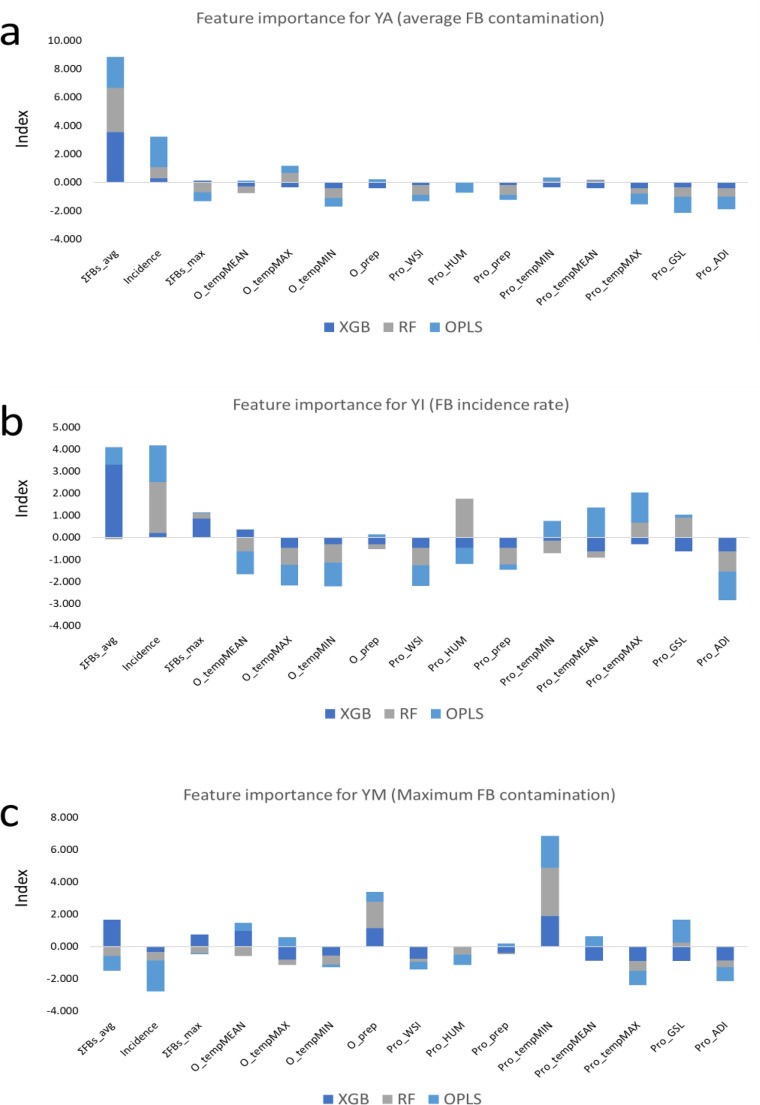
Variable importance plot.

The results of feature importance varied as different models showed varying feature significance in the modelling of the data (Fig. 3). Overall, the most important predictor in modelling of YA was ΣFBs_avg, which is the historical trend of average FB contamination in maize in the region (i.e., South Africa). The next most important feature was the incidence rate then followed by length of growing season. With regards to the YI response variable, FB incidence rate was the most important feature followed by ΣFBs_avg, and then humidity (Pro_Hum). Pro_tempMIN, O_prep, ΣFBs_avg, Incidence, and Pro_GSL were the five most important features in descending order of importance in modelling the patterns in YM.

## Discussion

South Africa is one of the most susceptible countries to climatic perturbations and variability due to many factors including its geographic location, surrounding landscape/features, relatively large agricultural sector, and limited adaptability capacity. It has been established that climatic conditions under which crops are cultivated critically influence fungi proliferation and associated mycotoxin production^7,12^. Analysis of climatic patterns over 16 years in this study shows increasing average temperatures over the study period, which is consistent with published research within the region and contributes to the wider conversation surrounding climate change. Nyoni et al.^24^ reported a similar temperature trend (rate of + 0.02 °C/year) as observed in this study in parts of South Africa between 1950 and 2016. According to James and Washington^25^, majority of Africa warms faster than what is noted in other parts of the world. Morishima and Akasaka^26^ documented significant upward trends in annual mean temperature for Southern Africa, while Engelbrecht et al.^27^ projected that a majority of Southern Africa will experience a temperature rise between 4 and 6 °C by the end of the century under the A2 (poor mitigation) scenario. According to Field^28^, Van der Fels-Klerx et al.^29^ and IPCC^30^, the anticipated consequences of climate change encompass a rise in mean global air temperatures and alterations in the distribution of precipitation, but notably, an increase in weather variability, characterised by the occurrence of more severe events such as heat waves, droughts, and extreme precipitation. According to our findings, these increasing temperatures are not just a one-time occurrence but are expected to persist in the future. These findings make a valuable contribution to the growing body of information that supports the existence of global warming and its potential consequences for the climate and ecosystem of South Africa.

In this study, we observe a notable variability in precipitation trend compared to temperature patterns. Elsewhere, Maúre et al.^31^ noted that precipitation projections in Southern Africa are considerably more variable than temperature projections. Also, our observed overall downward trajectory for precipitation is consistent with published research. For example, analysis of the Southern African climate under different global warming levels (GWL) by Maúre et al.^31^ revealed a decrease in precipitation by up to 4 mm/day throughout the Limpopo Basin and smaller regions of the Zambezi Basin in Zambia, as well as parts of the Western Cape in South Africa (under the 1.5 °C GWL). Decreases in precipitation are accompanied by an increase in consecutive dry days and a decrease in consecutive wet days throughout the region. According to Alberts et al.^21^, some parts of South Africa are expected to witness drier conditions and increased evaporation rates resulting in more frequent and severe droughts, thus having a significant impact on the contamination of crops by various fungi including mycotoxin-producing fungi such as *F*. *verticillioides*, and related species.

Mycotoxins, particularly FBs, have been a major food and feed contaminant and critical health risk factor in South Africa for many decades. In this study, data on FB monitoring over the study period show clear climate-dependent trends, and a strong positive correlation is likewise established between FB contamination and temperature. Several studies have indicated a positive correlation between temperature and drier conditions and mycotoxin contamination, where higher temperatures and drier conditions were implicated in higher mycotoxin occurrence and contamination levels. Periods of high mycotoxin prevalence are frequently associated with robust metabolic activities of mycotoxigenic fungal species, hence, an increase in mycotoxin contamination is anticipated. The accumulation of mycotoxins in crops is mostly influenced by climatic conditions, such as temperatures and severe variations in rainfall or drought episodes^32^. In the study conducted between 2013 and 2017^33^, it was noted that there was year-to-year variation in the incidence of mycotoxins and the levels of contamination in maize. The concentration of mycotoxins exhibited year-to-year variability across multiple regions, with their levels being impacted by fluctuations in weather conditions. Akello et al.^34^ reported high prevalence of FB (≈ 90%) in Southern African maize with associated high contamination levels (up to 40,000 µg/kg, n = 800), while low FB incidence (3.3%, n = 60) in finger millet was associated with relatively low contamination levels (max: 1500 µg/kg). The year 2016 was particularly very hot, perhaps the hottest in nearly a century, and resulted in the *El*-*nino* drought episode in Southern Africa during which time, FB concentrations in maize were particularly high^33,35^, and there were significant agricultural losses in estimates of millions of US dollars in monetary terms^36^. In 2017, East Asian maize harvested had a significant increase in FB concentration as a result of elevated temperatures^33^.

Again, our observed inverse correlation between precipitation and FB contamination levels is expected, as studies have linked drier conditions (lower precipitation) to increased mycotoxin contamination^21,37,38^. As highlighted earlier in the results section, climatic trend in this study shows a reasonable inverse correlation between maximum FB concentration and both precipitation and length of growing season. In 2015, the concentrations of FBs in Central Europa were elevated as a result of elevated temperatures and less precipitation during the silking and pre-harvest stages of maize cultivation, surpassing the levels observed in previous years^33^. In a study by Akello et al.^34^, the hottest and driest regions of Zimbabwe in Southern Africa with annual precipitation between 450 and 600 mm had the greatest mycotoxin concentrations. In the same study, it was likewise observed that maize harvested after the 2015/2016 cropping season and which had received minimal rainfall (368 mm) contained higher AF contamination levels than those of maize harvested after the 2016/2017 cropping season (rainfall: 536 mm). Interestingly, the opposite trend was observed for FB, which had lower concentrations in 2015/2016 (mean: 1191 μg/kg) as compared to 2016/2017 (mean: 3609 μg/kg). In 2017, there was a notable increase in aflatoxin B1 levels across Southeast Asia, East Asia, and Central Asia compared to the period of 2013–2016. This was be attributed to the elevated rainfall and temperatures seen during the maize silking phase^33^. The elevated levels of deoxynivalenol (DON) and zearalenone in maize cultivated in Central and Southern Europe in 2014, as compared to the years 2013, 2015, 2016, and 2017, was attributed to the combination of rainfall and mild temperatures seen during the flowering and maturity phase^33^.

Our generalized machine learning models were able to learn complex relationships from the data and provide reasonable predictions of potential future FB contamination levels (mean and maximum concentrations) as well as contamination incidence rate in maize. In some regard, some of the variables modelled, in particular YM, demonstrated some level of unsystematic variability. This is not totally unusual as to some extent, prediction of natural processes can be challenging due to multiple confounding factors and influences, some of which are unknown or difficult to establish. In the same light, modelling and predictive estimation of mycotoxin contamination patterns can be challenging due to the plethora of possible confounding variables including crop disease, weather/climatic conditions, physical damage to crops, fungi strain pathogenicity, toxigenicity, competition with other microbes in the plant, and the impact of fungicides on toxin biosynthesis^7,32,39–41^. In fact, the majority of mycotoxin predictive models are empirical in nature due to the limited understanding of the underlying processes that link illness development and toxin generation to the environment^32^. As a result, the prediction of mycotoxin contamination patterns can sometimes yield variable results. For example, mycotoxin [DON produced by *Fusarium* sp.] predictive models by Van der Fels-Klerx^29^ showed that DON contamination of wheat cultivated in Europe was, in overall, expected to increase, albeit with large variations, and in some locations and years a decrease in DON contamination was expected. This highlights some of the limitations of the study, as some critical variables influencing mycotoxin production in crops were not monitored in this study.

Regarding important model features, it is deduced from our study that the historical pattern of FB contamination in maize in the region (i.e., incidence rate, YA, and ΣFBs_max), various temperature variables, precipitation, and growing season length, amongst other features are the most significant determining factors when predicting future mycotoxin contamination. Incidence rate is concerned with the frequency of occurrence of FBs in the crop over time and provides critical information on the risk and burden of the mycotoxin in the food commodity within the time frame. Studies have correlated higher incidence rates with higher mycotoxin contamination^42–44^, underscoring its significance as a predictive indicator in elucidating the temporal trends and potential future trajectories of FB contamination in agricultural commodities. In agreement with this, studies have also shown that fungal spores present in plant residue or debris of earlier harvests may remain dormant in the soil from harvest from one year to the next^45,46^. As such, appropriate management of previous harvest plant biomass/residue by removal, burial, burning, etc. could help reduce fungal infection in the field.

Also, in this study, the identification of growing season length and precipitation as important model predictors of FB contamination patterns is in line with established knowledge. In the study by Fels-Klerx et al.^47^, a climate-based predictive model was developed for the modelling and prediction of DON in northwestern Europe for the future period 2031–2050. The model received several input variables including climatic data, nonetheless, the final optimized model and selected variables included length of time between flowering and harvest and other parameters relating to temperature, humidity, and precipitation. The length of growing season is one of the indices used to assess ecosystem dynamics/ climate extremes and relates to the period during which weather conditions are favourable for plant growth and maturity^12,48,49^. In this study, GSL i.e., growing season length, has a direct effect on YI and YM, while having an inverse effect on YA. Given the accuracies of the YA models, it may be inferred that a shorter GSL leads to a higher YA. Shorter GSL may suggest diminished precipitation levels, potentially resulting in drought-induced stress on crops, making the crops more susceptible to contamination by mycotoxigenic fungi, fostering the proliferation of mycotoxins. In contrast, a longer GSL leads to an increased incidence rate of mycotoxins and a greater maximum contamination of FB. In this context, the observed outcome can be inferred as a longer duration of the growing season, allowing for more time for crop maturation and thus prolonged exposure to pests, diseases, physical damage, and mycotoxigenic fungi in the field, which may consequently increase mycotoxins proliferation in the crop. Adequate precipitation promotes the growth of fungi and their spores, whilst increased humidity facilitates metabolic processes and the dispersal of spores, fostering favourable conditions for fungal colonisation of crops, hence increasing the likelihood of mycotoxin contamination in agricultural products. Gbashi et al.^7^, Ngoko et al.^50^, and Udoh et al.^51^ observed that wetter conditions and higher humidity increases the risks of mycotoxin proliferation in food crops.

Overall, predictive models, such as those generalized herein, could provide early warning alerts and insights into possible mycotoxin contamination risks. Indeed, such knowledge is critical to assist policy makers and other relevant actors along the food supply chain in identifying regions at risk of mycotoxin exposure, aiding in preparedness to take evasive measures. For example, in a study on the application of AI in improving real-time decision-making for high-impact weather, McGovern et al.^52^ observed that 75% of the time, expert forecasters choose the AI-based forecast over human intuition, providing proof that AI-based forecasts can improve decision making. It is thus, impossible to overstate the importance of predictive models not only in addressing food safety and security, but with regards to other effects of climate change. Our research highlights the significance of climate change and mycotoxin surveillance, predictive modelling, and data sharing for the control of mycotoxins along the food supply chain.

## Methods

### Study area

**Figure 4:**
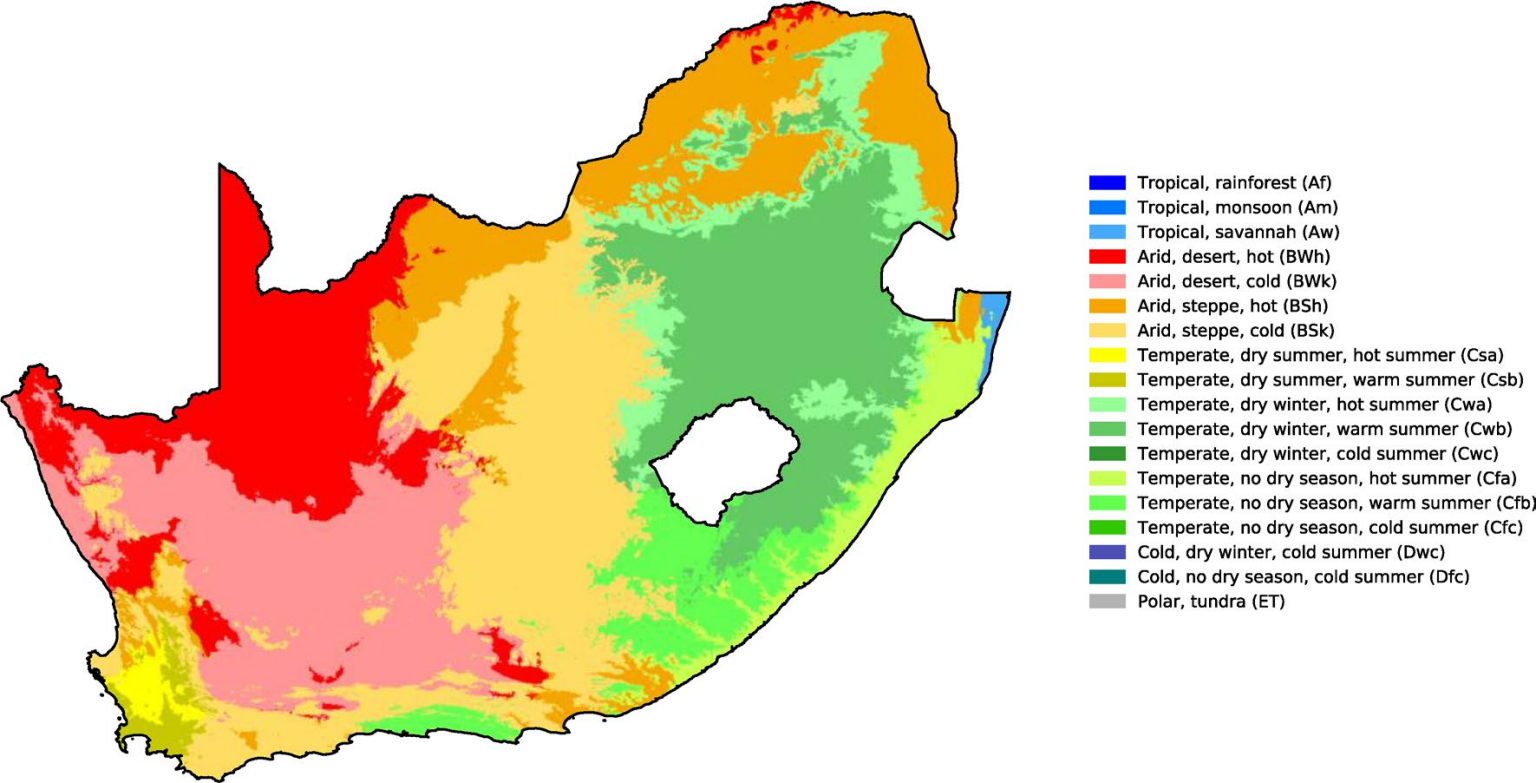
South Africa’s Köppen–Geiger climate classification (1980–2016)^54^.

South Africa is the southernmost country on the African continent and is located within latitude 30.559482° S and longitude 22.937506° E. It has the third largest biodiversity in the world. South Africa's many climatic zones (Fig. 4) are a result of the country's unique geography and the effect of the oceans. The climatic zones range from the harsh arid Namib desert in the country's extreme northwest to the lush subtropical environment between the Indian Ocean and Mozambique's borders in the east. Its agricultural sector is susceptible to numerous challenges, including erratic precipitation, mycotoxin contamination of crops, widespread poverty, environmental deterioration, and other comparable concerns^53^. As a result of its low adaptive capacity, South Africa's food system is very prone to the consequences of climate variability.

### Data on mycotoxin contamination

Data on FB contamination of maize in South Africa over the period between 2005/2006 and 2020/2021 (Supplementary 1), including average FB (ΣFBs_avg), maximum level (ΣFBs_max), and incidence rate (%) were retrieved from https://sagl.co.za/mycotoxin/ of the Southern African Grain Laboratory (SAGL) founded by the grain sector in Southern Africa in 1997 as an independent, non-profit laboratory to serve as a reference laboratory for grains and oil seeds. SAGL publishes data on major mycotoxins occurrence in important Southern African grains annually after appropriate sampling, sample preparation, analysis, and report generation. Details of the sample collection, preparation, analysis, and data generated are described by Meyer et al.^44^, with a brief description presented below.

In South Africa, maize is ideally sown in November or December and harvested in the following fall (autumn) i.e., April to May. When the crop is delivered by commercial farmers to commercial grain storage facilities, maize samples are taken for crop quality surveys from harvest to storage. After the grain handlers take a representative sample from each consignment for grading purposes, approximately 100 g of each sample is placed in corresponding bins (50–100 kg) as a subsample, in accordance with the class and grade assigned per silo bin at each silo. Each composite bin is thoroughly mixed before sending a 3-kg subsample to the laboratory for crop quality analysis. The received samples (i.e., about 1000 samples of white and yellow maize) are composite samples that represent maize produced in all of South Africa's commercially important producing regions by class and grade. For the mycotoxin analyses, approximately 35 to 40% of the survey samples from each season are (i.e., about 350 samples) are chosen to proportionally reflect all of the producing regions as well as white and yellow maize, each weighing 500 g each. An inhouse validated fit-for-purpose multi-mycotoxin analytical method at SAGL^44^, with acceptable method validation parameters is used for the analysis of the samples. The method is accredited for analysis of mycotoxins in cereals, grains, and related agricultural commodities based on the ISO 17025 by the South African National Accreditation System (SANAS)^44^. From the data generated, all results < LOD are reported as 0.

### South African climate

South African meteorological data between 2005 and 2021 retrieved from the World Bank Group, Climate Change Knowledge Portal (CCKP) (https://climateknowledgeportal.worldbank.org/download-data) was used. The dataset included Climate Research Unit (CRU)^55^ and Coupled Model Intercomparison Project Phase 6 (CMIP6)^56,57^ timeseries data on temperature, precipitation, humidity, length of growing season, and drought index. The CRU timeseries data is a very rich (high-resolution) climatic dataset with a 0.5° latitude by 0.5° longitude grid covering the entire world’s land domains with the exception of Antarctica. It is calculated by extrapolating monthly climatic anomalies from huge networks of weather station readings. For model training and forecasting, annual aggregates of these monthly data were retrieved from the database. Specific data features of CRU data retrieved included observed mean temperature (O_tempMEAN), observed maximum temperature (O_tempMAX), observed minimum temperature (O_tempMIN), and observed precipitation (O_prep). The CMIP6 dataset is generated using an ensemble of advanced computer algorithms, which simulate the earth's climate taking into account the interaction of many components, including the atmosphere, oceans, land, and ice. The CMIP framework was created to advance understanding of climate change, by using several models to fully explain past, current, and future climate changes brought about by natural, unforced variability or in response to changes in radiative forcings in a multi-model context^57^. Specific features of CMIP6 data retrieved included annual averages of the warm spell duration index (Pro_WSI), relative humidity (Pro_HUM), precipitation (Pro_prep), minimum temperature (Pro_tempMIN), mean temperature (Pro_tempMEAN), maximum temperature (Pro_tempMAX), growing season length (Pro_GSL), and annual standardized precipitation-evapotranspiration drought index (Pro_ADI).

### Data preparation

All the data were collated into a single spreadsheet and imported into Jupyter Notebook for processing. Missing data values were replaced by extrapolating adjacent values using the linear function of the Pandas library in order to fill in the void spaces based on the average of the preceding and succeeding values of the missing cell. The final data corpus comprised a multivariate time-series data structured with an annual time step. Data for SVM, XGB, and RF were featured-scaled to a range of − 1 to 1 using the Scikit-learn MinMax scaler algorithm. The retrieved mycotoxin contamination data were transformed by a one-step lag to generate the response variables, i.e., dependent variables, with YA = one-step lag of ΣFBs_avg, YI = one-step lag of Incidence, and YM = one-step lag of ΣFBs_max. Essentially, observations at prior time steps were used as inputs to predict the observation at the current time step.

### Descriptive and exploratory data analysis

Pearson’s bivariate correlation analysis was used to establish the direction and magnitude of the linear correlation between all pairs of variables studied. Augmented Dickey–Fuller (ADF) test was used to investigate the stationarity of each of the data variables over the study period. A stationary series is one whose values are not affected by the passage of time. As a result, the series' statistical characteristics, such as variance, mean, and autocorrelation, remains consistent over time. Thus, the ADF test provides insights into the trend patterns, stability, and variability of the variable with time. The ADF test uses an autoregressive model to investigate the unit root in the data. Autocorrelation of a series implies a correlation of the series with its previous values. The ADF test begins by posing the null hypothesis (Ho) that the time series is non-stationary and has a unit root. It then determines if the alternative hypothesis (Ha) that the given time series is stationary, should be statistically supported^58,59^ at *p* ≤ 0.05. The greater the negative value of the ADF statistic, the greater the level of confidence in rejecting the unit root hypothesis. The Granger causality test was used to test for Grangers causal interactions between variable pairs. The test is a probabilistic analysis, which determines whether past values of a time series contain information that helps to predict future values of another variable. The fundamental assumption is that if x causes y, then the prediction of y based on past values of y and past values of x should outperform (i.e., be more accurate than) the forecast of y based on past values of y alone^60^. The Ho is that the x series does not Granger cause the y series. If the Granger causality *p* value is less than or equal to the statistical significance level (0.05), then the null hypothesis is rejected in favour of the alternative hypothesis that the lag of x is critical in predicting y^60^.

### Predictive modelling using machine learning algorithms

Four different machine learning algorithms were trained on the historical mycotoxin and climatic data. The models adopted included SVM regressor, XGB regressor, RF regressor, and OPLS. The Sklearn implementation of SVM regressor algorithm in Python was used in this study. The model hyperparameters were optimized using grid search engine (GridSearchCV) by Sklearn in Python. The parameters optimized included kernel ('linear', 'rbf','poly'), C (100, 120, 150), gamma (1, 2, 5), and epsilon (1e−6, 1e−4, 1e−2, 1e−1, 1). Following the grid search optimization, the optimal hyperparameter used were: C: 25, epsilon: 1e-06, gamma: 5, and kernel: 'rbf'. The XGB model hyperparameters were optimized using similar approach described above. The parameters optimized included learning_rate (0.001, 0.01, 0.1, 1), n_estimators: (50, 100, 200, 300), and subsample (0.3, 0.5, 0.7, 1). The optimized conditions for YA and YI were learning_rate: 0.1, n_estimators: 50, and subsample: 0.3. The optimized conditions for YM were learning_rate: 0.01, n_estimators: 300, and subsample: 0.7. The Sklearn implementation of the RF algorithm in Python was used in this study. OPLS analysis was performed using SIMCA-P + 16.0 software (Umetrics, MKS Instruments Inc., Sweden) as described by Gbashi et al.^7^. For the analysis, the complete dataset was subjected to mean-centering and unit variate scaling prior to OPLS analysis. The data was categorized into X variables (predictors) and Y variables (responses). There were 3 responses and 15 predictors. The model adequacy was assessed by evaluation of the goodness-of-fit, goodness-of-prediction, and a number of other model fit parameters such as *R2*X(cum) and R2Y(cum).

The Kendall W test of concordance and the Friedman test were performed to interrogate the degree of agreement between raters (i.e., models) and to rank the efficacy of all employed models based on their performance as described by Mustapha and Saeed^61^. This test determines whether a group of raters arrive to equivalent rankings for a set of objects. In this study, the models, OPLS, SVM, RF, and XGB, and the model performance parameters (including RMSE, MAE, R^2^, and prediction residual) were used as the rank items. The output of this test is the Kendall concordance coefficient (Kendall W) and the associated probability significance level (*p* value). Kendall W is a number between 0 and 1 that represents agreement between raters (perfect agreement, Kendall W = 1). A significant Kendall *p* value implies a rejection of the null hypothesis (that performance among the models is due to chance or independent of one another), as such an overall ranking for the models can be given.

### Supplementary Information

Supplementary Information.

## Data Availability

Meteorological data utilized in this study are publicly available and accessible through the World Bank Group, Climate Change Knowledge Portal (CCKP) (https://climateknowledgeportal.worldbank.org/download-data). Data on mycotoxin (fumonisin) contamination of maize in South Africa utilized is also publicly available and accessible via the Southern African Grain Laboratory (SAGL) website (https://sagl.co.za/mycotoxin/). Researchers interested in accessing and analysing these data can obtain it directly from the specified repositories/sources. Additionally, for further assistance, specific instructions or requirements for accessing the data can be provided upon reasonable request from the corresponding author, [S.G.]. Data resulting from modelling and other statistical analysis in this study are available within the article [and its supplementary material] and may also be made available upon reasonable request from the corresponding author, [S.G.].

## Code availability

The code associated with the analyses presented in this study is written in the Python programming language (Python version 3.11.2) and is available upon reasonable request from the corresponding author, [S.G.]. Researchers seeking access to the code for replication, further exploration, or extension of the study findings are invited to contact the corresponding author for assistance.
