# Supplementary material for: Insights from modelling sixteen years of climatic and fumonisin patterns in maize in South Africa
Source: Sci Rep. 2024 May 21;14:11643. doi: 10.1038/s41598-024-60904-y (PMC11109125; doi:10.1038/s41598-024-60904-y)
Supplement: Supplementary file 1 — Supplementary Information. [file 41598_2024_60904_MOESM1_ESM.pdf]

Supplementary Materials

Supplementary Table 1: Augmented Dickey–Fuller (ADF) test of stationarity

**Variable**	**ADF Statistic**	**p-value**	**Critical Values (1%)**	**Critical Values (5%)**	**Critical Values (10%)**	**Comment**
ΣFBs_avg	6.106	1.000	-4.473	-3.290	-2.772	data has a unit root and is non-stationary
ΣFBs_max	0.586	0.987	-4.473	-3.290	-2.772	data has a unit root and is non-stationary
Pro_prep	0.452	0.983	-4.473	-3.290	-2.772	data has a unit root and is non-stationary
Pro_tempMIN	0.262	0.976	-4.223	-3.189	-2.730	data has a unit root and is non-stationary
O_tempMAX	0.134	0.968	-4.473	-3.290	-2.772	data has a unit root and is non-stationary
Pro_tempMAX	-0.140	0.945	-4.473	-3.290	-2.772	data has a unit root and is non-stationary
Incidence	-1.535	0.516	-4.473	-3.290	-2.772	data has a unit root and is non-stationary
Pro_tempMEAN	-2.096	0.246	-3.964	-3.085	-2.682	data has a unit root and is non-stationary
Pro_GSL	-2.230	0.196	-4.473	-3.290	-2.772	data has a unit root and is non-stationary
O_tempMIN	-2.342	0.159	-3.964	-3.085	-2.682	data has a unit root and is non-stationary
O_tempMEAN	-2.560	0.101	-3.964	-3.085	-2.682	data has a unit root and is non-stationary
Pro_ADI	-2.649	0.083	-3.964	-3.085	-2.682	data has a unit root and is non-stationary
O_prep	-3.312	0.014	-3.964	-3.085	-2.682	data does not have a unit root and is stationary
Pro_WSI	-3.422	0.010	-4.069	-3.127	-2.702	data does not have a unit root and is stationary
Pro_HUM	-3.581	0.006	-4.473	-3.290	-2.772	data does not have a unit root and is stationary

Supplementary Table 2: Grangers Causality Test

Variables	Lag	Stat	ssr_ftest	ssr_chi2test	lrtest	params_ftest
**ΣFBs_avg Vs Incidence**	**1**	F-value	0.513	0.641	0.628	0.513
	**1**	P-value	0.488	0.423	0.428	0.488
	**2**	F-value	0.764	2.377	2.195	0.764
	**2**	P-value	0.494	0.305	0.334	0.494
**Incidence Vs ΣFBs_avg**	**1**	F-value	0.107	0.134	0.134	0.107
	**1**	P-value	0.749	0.714	0.715	0.749
	**2**	F-value	0.215	0.668	0.652	0.215
	**2**	P-value	0.811	0.716	0.722	0.811
**ΣFBs_avg Vs ΣFBs_max**	**1**	F-value	2.170	2.712	2.493	2.170
	**1**	P-value	0.166	0.100	0.114	0.166
	**2**	F-value	1.084	3.373	3.022	1.084
	**2**	P-value	0.379	0.185	0.221	0.379
**ΣFBs_max Vs ΣFBs_avg**	**1**	F-value	0.274	0.343	0.339	0.274
	**1**	P-value	0.610	0.558	0.560	0.610
	**2**	F-value	0.555	1.725	1.627	0.555
	**2**	P-value	0.593	0.422	0.443	0.593
**ΣFBs_avg Vs O_tempMEAN**	**1**	F-value	0.820	1.025	0.992	0.820
	**1**	P-value	0.383	0.311	0.319	0.383
	**2**	F-value	0.712	2.214	2.055	0.712
	**2**	P-value	0.517	0.331	0.358	0.517
**O_tempMEAN Vs ΣFBs_avg**	**1**	F-value	0.000	0.000	0.000	0.000
	**1**	P-value	0.997	0.997	0.997	0.997
	**2**	F-value	0.830	2.583	2.370	0.830
	**2**	P-value	0.467	0.275	0.306	0.467
**ΣFBs_avg Vs O_tempMAX**	**1**	F-value	0.493	0.616	0.604	0.493
	**1**	P-value	0.496	0.432	0.437	0.496
	**2**	F-value	0.366	1.138	1.095	0.366
	**2**	P-value	0.703	0.566	0.579	0.703
**O_tempMAX Vs ΣFBs_avg**	**1**	F-value	0.066	0.082	0.082	0.066
	**1**	P-value	0.802	0.774	0.774	0.802
	**2**	F-value	1.323	4.116	3.608	1.323
	**2**	P-value	0.314	0.128	0.165	0.314
**ΣFBs_avg Vs O_tempMIN**	**1**	F-value	1.179	1.474	1.406	1.179
	**1**	P-value	0.299	0.225	0.236	0.299
	**2**	F-value	1.348	4.194	3.669	1.348
	**2**	P-value	0.308	0.123	0.160	0.308
**O_tempMIN Vs ΣFBs_avg**	**1**	F-value	0.068	0.084	0.084	0.068
	**1**	P-value	0.799	0.771	0.772	0.799
	**2**	F-value	0.333	1.035	0.998	0.333
	**2**	P-value	0.726	0.596	0.607	0.726
**ΣFBs_avg Vs O_prep**	**1**	F-value	0.334	0.418	0.412	0.334
	**1**	P-value	0.574	0.518	0.521	0.574
	**2**	F-value	1.343	4.178	3.656	1.343
	**2**	P-value	0.309	0.124	0.161	0.309

**O_prep Vs ΣFBs_avg**	**1**	F-value	0.172	0.215	0.214	0.172
	**1**	P-value	0.686	0.643	0.644	0.686
	**2**	F-value	0.688	2.142	1.993	0.688
	**2**	P-value	0.527	0.343	0.369	0.527
**ΣFBs_avg Vs Pro_WSI**	**1**	F-value	0.000	0.000	0.000	0.000
	**1**	P-value	0.998	0.997	0.997	0.998
	**2**	F-value	0.361	1.123	1.080	0.361
	**2**	P-value	0.707	0.570	0.583	0.707
**Pro_WSI Vs ΣFBs_avg**	**1**	F-value	0.138	0.173	0.172	0.138
	**1**	P-value	0.716	0.677	0.678	0.716
	**2**	F-value	0.036	0.113	0.112	0.036
	**2**	P-value	0.965	0.945	0.945	0.965
**ΣFBs_avg Vs Pro_HUM**	**1**	F-value	0.003	0.003	0.003	0.003
	**1**	P-value	0.960	0.954	0.954	0.960
	**2**	F-value	0.407	1.265	1.211	0.407
	**2**	P-value	0.678	0.531	0.546	0.678
**Pro_HUM Vs ΣFBs_avg**	**1**	F-value	0.221	0.277	0.274	0.221
	**1**	P-value	0.646	0.599	0.601	0.646
	**2**	F-value	0.542	1.687	1.593	0.542
	**2**	P-value	0.599	0.430	0.451	0.599
**ΣFBs_avg Vs Pro_prep**	**1**	F-value	1.101	1.376	1.317	1.101
	**1**	P-value	0.315	0.241	0.251	0.315
	**2**	F-value	0.636	1.978	1.851	0.636
	**2**	P-value	0.552	0.372	0.396	0.552
**Pro_prep Vs ΣFBs_avg**	**1**	F-value	0.005	0.007	0.007	0.005
	**1**	P-value	0.943	0.936	0.936	0.943
	**2**	F-value	0.674	2.098	1.955	0.674
	**2**	P-value	0.534	0.350	0.376	0.534
**ΣFBs_avg Vs Pro_tempMIN**	**1**	F-value	0.538	0.673	0.658	0.538
	**1**	P-value	0.477	0.412	0.417	0.477
	**2**	F-value	0.394	1.227	1.176	0.394
	**2**	P-value	0.685	0.541	0.555	0.685
**Pro_tempMIN Vs ΣFBs_avg**	**1**	F-value	1.243	1.553	1.478	1.243
	**1**	P-value	0.287	0.213	0.224	0.287
	**2**	F-value	1.144	3.558	3.170	1.144
	**2**	P-value	0.361	0.169	0.205	0.361
**ΣFBs_avg Vs Pro_tempMEAN**	**1**	F-value	0.339	0.424	0.418	0.339
	**1**	P-value	0.571	0.515	0.518	0.571
	**2**	F-value	0.549	1.709	1.612	0.549
	**2**	P-value	0.596	0.426	0.447	0.596
**Pro_tempMEAN Vs ΣFBs_avg**	**1**	F-value	0.883	1.104	1.065	0.883
	**1**	P-value	0.366	0.293	0.302	0.366
	**2**	F-value	0.524	1.631	1.543	0.524
	**2**	P-value	0.609	0.442	0.462	0.609
**ΣFBs_avg Vs Pro_tempMAX**	**1**	F-value	0.150	0.188	0.187	0.150
	**1**	P-value	0.705	0.665	0.666	0.705

	2	F-value	0.561	1.745	1.644	0.561
	2	P-value	0.589	0.418	0.439	0.589
**Pro_tempMAX Vs ΣFBs_avg**	1	F-value	1.023	1.279	1.227	1.023
	1	P-value	0.332	0.258	0.268	0.332
	2	F-value	0.842	2.620	2.402	0.842
	2	P-value	0.462	0.270	0.301	0.462
**ΣFBs_avg Vs Pro_GSL**	1	F-value	0.142	0.177	0.176	0.142
	1	P-value	0.713	0.674	0.675	0.713
	2	F-value	0.600	1.867	1.752	0.600
	2	P-value	0.569	0.393	0.416	0.569
**Pro_GSL Vs ΣFBs_avg**	1	F-value	0.108	0.136	0.135	0.108
	1	P-value	0.748	0.713	0.713	0.748
	2	F-value	0.622	1.935	1.812	0.622
	2	P-value	0.558	0.380	0.404	0.558
**ΣFBs_avg Vs Pro_ADI**	1	F-value	0.113	0.142	0.141	0.113
	1	P-value	0.742	0.707	0.707	0.742
	2	F-value	0.290	0.902	0.874	0.290
	2	P-value	0.755	0.637	0.646	0.755
**Pro_ADI Vs ΣFBs_avg**	1	F-value	0.452	0.566	0.555	0.452
	1	P-value	0.514	0.452	0.456	0.514
	2	F-value	0.299	0.930	0.900	0.299
	2	P-value	0.749	0.628	0.638	0.749
**Incidence Vs ΣFBs_max**	1	F-value	0.436	0.546	0.536	0.436
	1	P-value	0.521	0.460	0.464	0.521
	2	F-value	1.670	5.194	4.418	1.670
	2	P-value	0.242	0.074	0.110	0.242
**ΣFBs_max Vs Incidence**	1	F-value	0.002	0.002	0.002	0.002
	1	P-value	0.967	0.963	0.963	0.967
	2	F-value	0.135	0.420	0.414	0.135
	2	P-value	0.875	0.810	0.813	0.875
**Incidence Vs O_tempMEAN**	1	F-value	0.010	0.013	0.013	0.010
	1	P-value	0.921	0.910	0.910	0.921
	2	F-value	2.047	6.368	5.249	2.047
	2	P-value	0.185	0.041	0.072	0.185
**O_tempMEAN Vs Incidence**	1	F-value	0.255	0.319	0.316	0.255
	1	P-value	0.622	0.572	0.574	0.622
	2	F-value	0.600	1.867	1.753	0.600
	2	P-value	0.569	0.393	0.416	0.569
**Incidence Vs O_tempMAX**	1	F-value	0.072	0.090	0.090	0.072
	1	P-value	0.793	0.764	0.764	0.793
	2	F-value	1.050	3.266	2.936	1.050
	2	P-value	0.389	0.195	0.230	0.389
**O_tempMAX Vs Incidence**	1	F-value	0.214	0.267	0.265	0.214
	1	P-value	0.652	0.605	0.607	0.652
	2	F-value	0.375	1.168	1.122	0.375
	2	P-value	0.697	0.558	0.571	0.697

**Incidence Vs O_tempMIN**	**1**	F-value	0.458	0.572	0.561	0.458
	**1**	P-value	0.512	0.449	0.454	0.512
	**2**	F-value	4.131	12.853	9.119	4.131
	**2**	P-value	0.053	0.002	0.010	0.053
**O_tempMIN Vs Incidence**	**1**	F-value	0.227	0.283	0.281	0.227
	**1**	P-value	0.642	0.594	0.596	0.642
	**2**	F-value	1.140	3.547	3.162	1.140
	**2**	P-value	0.362	0.170	0.206	0.362
**Incidence Vs O_prep**	**1**	F-value	2.130	2.662	2.451	2.130
	**1**	P-value	0.170	0.103	0.117	0.170
	**2**	F-value	1.484	4.617	3.990	1.484
	**2**	P-value	0.277	0.099	0.136	0.277
**O_prep Vs Incidence**	**1**	F-value	1.001	1.251	1.201	1.001
	**1**	P-value	0.337	0.263	0.273	0.337
	**2**	F-value	0.054	0.168	0.167	0.054
	**2**	P-value	0.948	0.919	0.920	0.948
**Incidence Vs Pro_WSI**	**1**	F-value	0.000	0.000	0.000	0.000
	**1**	P-value	0.991	0.990	0.990	0.991
	**2**	F-value	2.067	6.430	5.291	2.067
	**2**	P-value	0.183	0.040	0.071	0.183
**Pro_WSI Vs Incidence**	**1**	F-value	0.177	0.221	0.219	0.177
	**1**	P-value	0.682	0.639	0.640	0.682
	**2**	F-value	2.879	8.958	6.925	2.879
	**2**	P-value	0.108	0.011	0.031	0.108
**Incidence Vs Pro_HUM**	**1**	F-value	2.265	2.832	2.594	2.265
	**1**	P-value	0.158	0.092	0.107	0.158
	**2**	F-value	0.740	2.303	2.132	0.740
	**2**	P-value	0.504	0.316	0.344	0.504
**Pro_HUM Vs Incidence**	**1**	F-value	0.080	0.100	0.100	0.080
	**1**	P-value	0.782	0.751	0.752	0.782
	**2**	F-value	0.449	1.396	1.330	0.449
	**2**	P-value	0.652	0.498	0.514	0.652
**Incidence Vs Pro_prep**	**1**	F-value	2.842	3.553	3.189	2.842
	**1**	P-value	0.118	0.059	0.074	0.118
	**2**	F-value	3.194	9.937	7.509	3.194
	**2**	P-value	0.089	0.007	0.023	0.089
**Pro_prep Vs Incidence**	**1**	F-value	1.379	1.724	1.632	1.379
	**1**	P-value	0.263	0.189	0.201	0.263
	**2**	F-value	0.770	2.395	2.211	0.770
	**2**	P-value	0.491	0.302	0.331	0.491
**Incidence Vs Pro_tempMIN**	**1**	F-value	0.132	0.165	0.164	0.132
	**1**	P-value	0.723	0.685	0.685	0.723
	**2**	F-value	0.094	0.293	0.290	0.094
	**2**	P-value	0.911	0.864	0.865	0.911
**Pro_tempMIN Vs Incidence**	**1**	F-value	0.068	0.085	0.085	0.068
	**1**	P-value	0.799	0.771	0.771	0.799

	2	F-value	1.814	5.644	4.742	1.814
	2	P-value	0.218	0.059	0.093	0.218
**Incidence Vs Pro_tempMEAN**	1	F-value	0.231	0.289	0.286	0.231
	1	P-value	0.639	0.591	0.593	0.639
	2	F-value	0.430	1.338	1.277	0.430
	2	P-value	0.663	0.512	0.528	0.663
**Pro_tempMEAN Vs Incidence**	1	F-value	0.217	0.272	0.269	0.217
	1	P-value	0.649	0.602	0.604	0.649
	2	F-value	0.886	2.757	2.517	0.886
	2	P-value	0.445	0.252	0.284	0.445
**Incidence Vs Pro_tempMAX**	1	F-value	0.378	0.472	0.465	0.378
	1	P-value	0.550	0.492	0.495	0.550
	2	F-value	1.561	4.856	4.169	1.561
	2	P-value	0.262	0.088	0.124	0.262
**Pro_tempMAX Vs Incidence**	1	F-value	0.724	0.905	0.879	0.724
	1	P-value	0.412	0.341	0.349	0.412
	2	F-value	0.700	2.177	2.023	0.700
	2	P-value	0.522	0.337	0.364	0.522
**Incidence Vs Pro_GSL**	1	F-value	0.233	0.291	0.288	0.233
	1	P-value	0.638	0.590	0.591	0.638
	2	F-value	0.705	2.194	2.038	0.705
	2	P-value	0.519	0.334	0.361	0.519
**Pro_GSL Vs Incidence**	1	F-value	0.003	0.004	0.004	0.003
	1	P-value	0.956	0.950	0.950	0.956
	2	F-value	4.390	13.656	9.531	4.390
	2	P-value	0.047	0.001	0.009	0.047
**Incidence Vs Pro_ADI**	1	F-value	0.256	0.320	0.317	0.256
	1	P-value	0.622	0.571	0.573	0.622
	2	F-value	0.000	0.000	0.000	0.000
	2	P-value	1.000	1.000	1.000	1.000
**Pro_ADI Vs Incidence**	1	F-value	0.166	0.207	0.206	0.166
	1	P-value	0.691	0.649	0.650	0.691
	2	F-value	1.367	4.252	3.713	1.367
	2	P-value	0.303	0.119	0.156	0.303
**ΣFBs_max Vs O_tempMEAN**	1	F-value	0.962	1.202	1.157	0.962
	1	P-value	0.346	0.273	0.282	0.346
	2	F-value	0.353	1.097	1.056	0.353
	2	P-value	0.712	0.578	0.590	0.712
**O_tempMEAN Vs ΣFBs_max**	1	F-value	0.829	1.036	1.002	0.829
	1	P-value	0.380	0.309	0.317	0.380
	2	F-value	0.533	1.659	1.568	0.533
	2	P-value	0.604	0.436	0.457	0.604
**ΣFBs_max Vs O_tempMAX**	1	F-value	1.362	1.703	1.613	1.362
	1	P-value	0.266	0.192	0.204	0.266
	2	F-value	0.711	2.211	2.053	0.711
	2	P-value	0.517	0.331	0.358	0.517

**O_tempMAX Vs ΣFBs_max**	**1**	F-value	1.262	1.577	1.500	1.262
	**1**	P-value	0.283	0.209	0.221	0.283
	**2**	F-value	1.599	4.974	4.256	1.599
	**2**	P-value	0.255	0.083	0.119	0.255
**ΣFBs_max Vs O_tempMIN**	**1**	F-value	0.231	0.289	0.286	0.231
	**1**	P-value	0.639	0.591	0.593	0.639
	**2**	F-value	0.142	0.443	0.436	0.142
	**2**	P-value	0.869	0.801	0.804	0.869
**O_tempMIN Vs ΣFBs_max**	**1**	F-value	0.125	0.157	0.156	0.125
	**1**	P-value	0.729	0.692	0.693	0.729
	**2**	F-value	0.192	0.598	0.586	0.192
	**2**	P-value	0.828	0.741	0.746	0.828
**ΣFBs_max Vs O_prep**	**1**	F-value	3.425	4.281	3.766	3.425
	**1**	P-value	0.089	0.039	0.052	0.089
	**2**	F-value	1.938	6.030	5.014	1.938
	**2**	P-value	0.200	0.049	0.081	0.200
**O_prep Vs ΣFBs_max**	**1**	F-value	0.144	0.180	0.179	0.144
	**1**	P-value	0.711	0.671	0.672	0.711
	**2**	F-value	0.854	2.658	2.434	0.854
	**2**	P-value	0.457	0.265	0.296	0.457
**ΣFBs_max Vs Pro_WSI**	**1**	F-value	1.287	1.609	1.529	1.287
	**1**	P-value	0.279	0.205	0.216	0.279
	**2**	F-value	4.518	14.056	9.732	4.518
	**2**	P-value	0.044	0.001	0.008	0.044
**Pro_WSI Vs ΣFBs_max**	**1**	F-value	2.632	3.289	2.974	2.632
	**1**	P-value	0.131	0.070	0.085	0.131
	**2**	F-value	2.068	6.435	5.295	2.068
	**2**	P-value	0.182	0.040	0.071	0.182
**ΣFBs_max Vs Pro_HUM**	**1**	F-value	0.026	0.032	0.032	0.026
	**1**	P-value	0.875	0.858	0.858	0.875
	**2**	F-value	0.053	0.165	0.164	0.053
	**2**	P-value	0.949	0.921	0.921	0.949
**Pro_HUM Vs ΣFBs_max**	**1**	F-value	7.674	9.593	7.416	7.674
	**1**	P-value	0.017	0.002	0.006	0.017
	**2**	F-value	2.980	9.270	7.113	2.980
	**2**	P-value	0.102	0.010	0.029	0.102
**ΣFBs_max Vs Pro_prep**	**1**	F-value	2.124	2.655	2.445	2.124
	**1**	P-value	0.171	0.103	0.118	0.171
	**2**	F-value	2.670	8.307	6.522	2.670
	**2**	P-value	0.123	0.016	0.038	0.123
**Pro_prep Vs ΣFBs_max**	**1**	F-value	1.121	1.401	1.339	1.121
	**1**	P-value	0.311	0.237	0.247	0.311
	**2**	F-value	0.374	1.164	1.118	0.374
	**2**	P-value	0.698	0.559	0.572	0.698
**ΣFBs_max Vs Pro_tempMIN**	**1**	F-value	2.197	2.746	2.521	2.197
	**1**	P-value	0.164	0.098	0.112	0.164

	2	F-value	1.989	6.187	5.124	1.989
	2	P-value	0.193	0.045	0.077	0.193
**Pro_tempMIN Vs ΣFBs_max**	1	F-value	13.145	16.431	11.096	13.145
	1	P-value	0.003	0.000	0.001	0.003
	2	F-value	1.856	5.775	4.835	1.856
	2	P-value	0.211	0.056	0.089	0.211
**ΣFBs_max Vs Pro_tempMEAN**	1	F-value	0.553	0.691	0.675	0.553
	1	P-value	0.472	0.406	0.411	0.472
	2	F-value	0.608	1.891	1.773	0.608
	2	P-value	0.566	0.389	0.412	0.566
**Pro_tempMEAN Vs ΣFBs_max**	1	F-value	29.373	36.717	18.566	29.373
	1	P-value	0.000	0.000	0.000	0.000
	2	F-value	9.267	28.832	15.655	9.267
	2	P-value	0.007	0.000	0.000	0.007
**ΣFBs_max Vs Pro_tempMAX**	1	F-value	0.154	0.192	0.191	0.154
	1	P-value	0.702	0.661	0.662	0.702
	2	F-value	0.163	0.506	0.497	0.163
	2	P-value	0.852	0.776	0.780	0.852
**Pro_tempMAX Vs ΣFBs_max**	1	F-value	25.400	31.750	17.051	25.400
	1	P-value	0.000	0.000	0.000	0.000
	2	F-value	16.156	50.264	21.335	16.156
	2	P-value	0.001	0.000	0.000	0.001
**ΣFBs_max Vs Pro_GSL**	1	F-value	3.320	4.150	3.664	3.320
	1	P-value	0.093	0.042	0.056	0.093
	2	F-value	4.110	12.785	9.083	4.110
	2	P-value	0.054	0.002	0.011	0.054
**Pro_GSL Vs ΣFBs_max**	1	F-value	0.176	0.220	0.218	0.176
	1	P-value	0.682	0.639	0.640	0.682
	2	F-value	0.247	0.769	0.749	0.247
	2	P-value	0.786	0.681	0.688	0.786
**ΣFBs_max Vs Pro_ADI**	1	F-value	0.066	0.082	0.082	0.066
	1	P-value	0.802	0.774	0.774	0.802
	2	F-value	1.084	3.372	3.021	1.084
	2	P-value	0.379	0.185	0.221	0.379
**Pro_ADI Vs ΣFBs_max**	1	F-value	1.589	1.986	1.865	1.589
	1	P-value	0.231	0.159	0.172	0.231
	2	F-value	1.023	3.182	2.867	1.023
	2	P-value	0.398	0.204	0.238	0.398
**O_tempMEAN Vs O_tempMAX**	1	F-value	0.272	0.340	0.336	0.272
	1	P-value	0.611	0.560	0.562	0.611
	2	F-value	1.254	3.902	3.442	1.254
	2	P-value	0.331	0.142	0.179	0.331
**O_tempMAX Vs O_tempMEAN**	1	F-value	0.244	0.304	0.301	0.244
	1	P-value	0.631	0.581	0.583	0.631
	2	F-value	1.288	4.007	3.524	1.288
	2	P-value	0.322	0.135	0.172	0.322

**O_tempMEAN Vs O_tempMIN**	**1**	F-value	0.221	0.277	0.274	0.221
	**1**	P-value	0.647	0.599	0.601	0.647
	**2**	F-value	1.551	4.825	4.146	1.551
	**2**	P-value	0.264	0.090	0.126	0.264
**O_tempMIN Vs O_tempMEAN**	**1**	F-value	0.341	0.426	0.420	0.341
	**1**	P-value	0.570	0.514	0.517	0.570
	**2**	F-value	0.447	1.390	1.325	0.447
	**2**	P-value	0.653	0.499	0.515	0.653
**O_tempMEAN Vs O_prep**	**1**	F-value	4.861	6.076	5.102	4.861
	**1**	P-value	0.048	0.014	0.024	0.048
	**2**	F-value	4.122	12.825	9.104	4.122
	**2**	P-value	0.054	0.002	0.011	0.054
**O_prep Vs O_tempMEAN**	**1**	F-value	1.058	1.322	1.267	1.058
	**1**	P-value	0.324	0.250	0.260	0.324
	**2**	F-value	0.842	2.620	2.402	0.842
	**2**	P-value	0.462	0.270	0.301	0.462
**O_tempMEAN Vs Pro_WSI**	**1**	F-value	0.437	0.546	0.536	0.437
	**1**	P-value	0.521	0.460	0.464	0.521
	**2**	F-value	0.369	1.147	1.103	0.369
	**2**	P-value	0.702	0.564	0.576	0.702
**Pro_WSI Vs O_tempMEAN**	**1**	F-value	0.720	0.900	0.874	0.720
	**1**	P-value	0.413	0.343	0.350	0.413
	**2**	F-value	0.712	2.216	2.057	0.712
	**2**	P-value	0.516	0.330	0.358	0.516
**O_tempMEAN Vs Pro_HUM**	**1**	F-value	0.333	0.417	0.411	0.333
	**1**	P-value	0.574	0.519	0.522	0.574
	**2**	F-value	0.417	1.299	1.242	0.417
	**2**	P-value	0.671	0.522	0.537	0.671
**Pro_HUM Vs O_tempMEAN**	**1**	F-value	0.307	0.384	0.379	0.307
	**1**	P-value	0.590	0.535	0.538	0.590
	**2**	F-value	0.182	0.567	0.556	0.182
	**2**	P-value	0.836	0.753	0.757	0.836
**O_tempMEAN Vs Pro_prep**	**1**	F-value	2.416	3.019	2.751	2.416
	**1**	P-value	0.146	0.082	0.097	0.146
	**2**	F-value	1.240	3.858	3.408	1.240
	**2**	P-value	0.334	0.145	0.182	0.334
**Pro_prep Vs O_tempMEAN**	**1**	F-value	0.283	0.354	0.350	0.283
	**1**	P-value	0.604	0.552	0.554	0.604
	**2**	F-value	1.488	4.628	3.998	1.488
	**2**	P-value	0.277	0.099	0.135	0.277
**O_tempMEAN Vs Pro_tempMIN**	**1**	F-value	0.673	0.841	0.818	0.673
	**1**	P-value	0.428	0.359	0.366	0.428
	**2**	F-value	1.031	3.209	2.889	1.031
	**2**	P-value	0.395	0.201	0.236	0.395
	**1**	F-value	4.895	6.119	5.132	4.895
	**1**	P-value	0.047	0.013	0.023	0.047

**Pro_tempMIN Vs O_tempMEAN**	**2**	F-value	0.021	0.064	0.064	0.021
	**2**	P-value	0.980	0.968	0.969	0.980
**O_tempMEAN Vs Pro_tempMEAN**	**1**	F-value	0.134	0.167	0.166	0.134
	**1**	P-value	0.721	0.682	0.683	0.721
	**2**	F-value	0.424	1.320	1.262	0.424
	**2**	P-value	0.667	0.517	0.532	0.667
**Pro_tempMEAN Vs O_tempMEAN**	**1**	F-value	4.160	5.200	4.464	4.160
	**1**	P-value	0.064	0.023	0.035	0.064
	**2**	F-value	0.762	2.370	2.190	0.762
	**2**	P-value	0.495	0.306	0.335	0.495
**O_tempMEAN Vs Pro_tempMAX**	**1**	F-value	0.018	0.022	0.022	0.018
	**1**	P-value	0.897	0.882	0.882	0.897
	**2**	F-value	0.332	1.033	0.996	0.332
	**2**	P-value	0.726	0.597	0.608	0.726
**Pro_tempMAX Vs O_tempMEAN**	**1**	F-value	2.834	3.542	3.180	2.834
	**1**	P-value	0.118	0.060	0.075	0.118
	**2**	F-value	1.554	4.834	4.152	1.554
	**2**	P-value	0.263	0.089	0.125	0.263
**O_tempMEAN Vs Pro_GSL**	**1**	F-value	1.149	1.436	1.372	1.149
	**1**	P-value	0.305	0.231	0.242	0.305
	**2**	F-value	1.025	3.188	2.872	1.025
	**2**	P-value	0.397	0.203	0.238	0.397
**Pro_GSL Vs O_tempMEAN**	**1**	F-value	2.591	3.239	2.933	2.591
	**1**	P-value	0.133	0.072	0.087	0.133
	**2**	F-value	1.453	4.522	3.919	1.453
	**2**	P-value	0.284	0.104	0.141	0.284
**O_tempMEAN Vs Pro_ADI**	**1**	F-value	0.113	0.142	0.141	0.113
	**1**	P-value	0.742	0.707	0.707	0.742
	**2**	F-value	0.737	2.293	2.124	0.737
	**2**	P-value	0.505	0.318	0.346	0.505
**Pro_ADI Vs O_tempMEAN**	**1**	F-value	0.649	0.812	0.791	0.649
	**1**	P-value	0.436	0.368	0.374	0.436
	**2**	F-value	0.393	1.224	1.173	0.393
	**2**	P-value	0.686	0.542	0.556	0.686
**O_tempMAX Vs O_tempMIN**	**1**	F-value	0.217	0.271	0.269	0.217
	**1**	P-value	0.650	0.603	0.604	0.650
	**2**	F-value	1.471	4.577	3.960	1.471
	**2**	P-value	0.280	0.101	0.138	0.280
**O_tempMIN Vs O_tempMAX**	**1**	F-value	0.369	0.461	0.454	0.369
	**1**	P-value	0.555	0.497	0.501	0.555
	**2**	F-value	0.400	1.244	1.192	0.400
	**2**	P-value	0.682	0.537	0.551	0.682
**O_tempMAX Vs O_prep**	**1**	F-value	2.239	2.799	2.567	2.239
	**1**	P-value	0.160	0.094	0.109	0.160
	**2**	F-value	1.688	5.252	4.460	1.688
	**2**	P-value	0.238	0.072	0.108	0.238

**O_prep Vs O_tempMAX**	**1**	F-value	0.393	0.491	0.483	0.393
	**1**	P-value	0.543	0.484	0.487	0.543
	**2**	F-value	0.274	0.852	0.827	0.274
	**2**	P-value	0.767	0.653	0.661	0.767
**O_tempMAX Vs Pro_WSI**	**1**	F-value	0.302	0.378	0.373	0.302
	**1**	P-value	0.593	0.539	0.541	0.593
	**2**	F-value	0.112	0.347	0.343	0.112
	**2**	P-value	0.896	0.841	0.842	0.896
**Pro_WSI Vs O_tempMAX**	**1**	F-value	0.448	0.560	0.549	0.448
	**1**	P-value	0.516	0.454	0.459	0.516
	**2**	F-value	0.756	2.353	2.175	0.756
	**2**	P-value	0.497	0.308	0.337	0.497
**O_tempMAX Vs Pro_HUM**	**1**	F-value	0.696	0.870	0.845	0.696
	**1**	P-value	0.421	0.351	0.358	0.421
	**2**	F-value	0.524	1.631	1.542	0.524
	**2**	P-value	0.609	0.442	0.462	0.609
**Pro_HUM Vs O_tempMAX**	**1**	F-value	0.208	0.260	0.258	0.208
	**1**	P-value	0.657	0.610	0.612	0.657
	**2**	F-value	0.227	0.706	0.689	0.227
	**2**	P-value	0.801	0.703	0.709	0.801
**O_tempMAX Vs Pro_prep**	**1**	F-value	3.201	4.001	3.547	3.201
	**1**	P-value	0.099	0.045	0.060	0.099
	**2**	F-value	1.369	4.258	3.718	1.369
	**2**	P-value	0.303	0.119	0.156	0.303
**Pro_prep Vs O_tempMAX**	**1**	F-value	0.094	0.117	0.117	0.094
	**1**	P-value	0.765	0.732	0.733	0.765
	**2**	F-value	1.769	5.503	4.641	1.769
	**2**	P-value	0.225	0.064	0.098	0.225
**O_tempMAX Vs Pro_tempMIN**	**1**	F-value	1.274	1.592	1.513	1.274
	**1**	P-value	0.281	0.207	0.219	0.281
	**2**	F-value	0.846	2.632	2.412	0.846
	**2**	P-value	0.461	0.268	0.299	0.461
**Pro_tempMIN Vs O_tempMAX**	**1**	F-value	7.019	8.773	6.908	7.019
	**1**	P-value	0.021	0.003	0.009	0.021
	**2**	F-value	0.318	0.989	0.955	0.318
	**2**	P-value	0.736	0.610	0.620	0.736
**O_tempMAX Vs Pro_tempMEAN**	**1**	F-value	0.296	0.370	0.366	0.296
	**1**	P-value	0.596	0.543	0.545	0.596
	**2**	F-value	0.593	1.845	1.733	0.593
	**2**	P-value	0.573	0.398	0.420	0.573
**Pro_tempMEAN Vs O_tempMAX**	**1**	F-value	5.756	7.195	5.878	5.756
	**1**	P-value	0.034	0.007	0.015	0.034
	**2**	F-value	1.786	5.556	4.679	1.786
	**2**	P-value	0.222	0.062	0.096	0.222
**O_tempMAX Vs Pro_tempMAX**	**1**	F-value	0.084	0.106	0.105	0.084
	**1**	P-value	0.776	0.745	0.746	0.776

	2	F-value	0.752	2.339	2.163	0.752
	2	P-value	0.499	0.311	0.339	0.499
**Pro_tempMAX Vs O_tempMAX**	1	F-value	3.835	4.793	4.159	3.835
	1	P-value	0.074	0.029	0.041	0.074
	2	F-value	2.144	6.670	5.455	2.144
	2	P-value	0.173	0.036	0.065	0.173
**O_tempMAX Vs Pro_GSL**	1	F-value	1.146	1.433	1.368	1.146
	1	P-value	0.305	0.231	0.242	0.305
	2	F-value	0.484	1.507	1.431	0.484
	2	P-value	0.631	0.471	0.489	0.631
**Pro_GSL Vs O_tempMAX**	1	F-value	2.525	3.156	2.865	2.525
	1	P-value	0.138	0.076	0.091	0.138
	2	F-value	1.066	3.316	2.976	1.066
	2	P-value	0.384	0.191	0.226	0.384
**O_tempMAX Vs Pro_ADI**	1	F-value	0.487	0.608	0.596	0.487
	1	P-value	0.499	0.435	0.440	0.499
	2	F-value	0.400	1.245	1.193	0.400
	2	P-value	0.682	0.537	0.551	0.682
**Pro_ADI Vs O_tempMAX**	1	F-value	0.542	0.678	0.663	0.542
	1	P-value	0.476	0.410	0.415	0.476
	2	F-value	0.376	1.169	1.123	0.376
	2	P-value	0.697	0.557	0.570	0.697
**O_tempMIN Vs O_prep**	1	F-value	6.605	8.256	6.578	6.605
	1	P-value	0.025	0.004	0.010	0.025
	2	F-value	2.788	8.672	6.749	2.788
	2	P-value	0.114	0.013	0.034	0.114
**O_prep Vs O_tempMIN**	1	F-value	1.619	2.023	1.898	1.619
	1	P-value	0.227	0.155	0.168	0.227
	2	F-value	2.088	6.497	5.337	2.088
	2	P-value	0.180	0.039	0.069	0.180
**O_tempMIN Vs Pro_WSI**	1	F-value	0.572	0.715	0.699	0.572
	1	P-value	0.464	0.398	0.403	0.464
	2	F-value	1.510	4.697	4.050	1.510
	2	P-value	0.272	0.096	0.132	0.272
**Pro_WSI Vs O_tempMIN**	1	F-value	0.778	0.973	0.943	0.778
	1	P-value	0.395	0.324	0.332	0.395
	2	F-value	0.332	1.034	0.998	0.332
	2	P-value	0.726	0.596	0.607	0.726
**O_tempMIN Vs Pro_HUM**	1	F-value	0.030	0.038	0.038	0.030
	1	P-value	0.865	0.846	0.846	0.865
	2	F-value	0.540	1.681	1.588	0.540
	2	P-value	0.600	0.431	0.452	0.600
**Pro_HUM Vs O_tempMIN**	1	F-value	0.420	0.525	0.516	0.420
	1	P-value	0.529	0.469	0.472	0.529
	2	F-value	0.160	0.499	0.490	0.160
	2	P-value	0.854	0.779	0.783	0.854

**O_tempMIN Vs Pro_prep**	**1**	F-value	1.283	1.604	1.524	1.283
	**1**	P-value	0.279	0.205	0.217	0.279
	**2**	F-value	1.164	3.623	3.222	1.164
	**2**	P-value	0.355	0.163	0.200	0.355
**Pro_prep Vs O_tempMIN**	**1**	F-value	0.535	0.668	0.654	0.535
	**1**	P-value	0.479	0.414	0.419	0.479
	**2**	F-value	0.746	2.320	2.147	0.746
	**2**	P-value	0.502	0.314	0.342	0.502
**O_tempMIN Vs Pro_tempMIN**	**1**	F-value	0.121	0.152	0.151	0.121
	**1**	P-value	0.734	0.697	0.698	0.734
	**2**	F-value	0.450	1.401	1.336	0.450
	**2**	P-value	0.651	0.496	0.513	0.651
**Pro_tempMIN Vs O_tempMIN**	**1**	F-value	1.520	1.900	1.789	1.520
	**1**	P-value	0.241	0.168	0.181	0.241
	**2**	F-value	0.222	0.691	0.675	0.222
	**2**	P-value	0.805	0.708	0.714	0.805
**O_tempMIN Vs Pro_tempMEAN**	**1**	F-value	0.009	0.011	0.011	0.009
	**1**	P-value	0.927	0.916	0.917	0.927
	**2**	F-value	0.181	0.562	0.551	0.181
	**2**	P-value	0.838	0.755	0.759	0.838
**Pro_tempMEAN Vs O_tempMIN**	**1**	F-value	1.459	1.824	1.721	1.459
	**1**	P-value	0.250	0.177	0.190	0.250
	**2**	F-value	0.156	0.486	0.477	0.156
	**2**	P-value	0.858	0.784	0.788	0.858
**O_tempMIN Vs Pro_tempMAX**	**1**	F-value	0.009	0.012	0.012	0.009
	**1**	P-value	0.924	0.914	0.914	0.924
	**2**	F-value	0.062	0.193	0.192	0.062
	**2**	P-value	0.940	0.908	0.908	0.940
**Pro_tempMAX Vs O_tempMIN**	**1**	F-value	1.045	1.307	1.253	1.045
	**1**	P-value	0.327	0.253	0.263	0.327
	**2**	F-value	0.696	2.164	2.012	0.696
	**2**	P-value	0.524	0.339	0.366	0.524
**O_tempMIN Vs Pro_GSL**	**1**	F-value	0.961	1.201	1.155	0.961
	**1**	P-value	0.346	0.273	0.282	0.346
	**2**	F-value	1.762	5.481	4.625	1.762
	**2**	P-value	0.226	0.065	0.099	0.226
**Pro_GSL Vs O_tempMIN**	**1**	F-value	1.603	2.004	1.881	1.603
	**1**	P-value	0.229	0.157	0.170	0.229
	**2**	F-value	1.371	4.266	3.724	1.371
	**2**	P-value	0.302	0.118	0.155	0.302
**O_tempMIN Vs Pro_ADI**	**1**	F-value	0.019	0.023	0.023	0.019
	**1**	P-value	0.894	0.879	0.879	0.894
	**2**	F-value	1.957	6.089	5.056	1.957
	**2**	P-value	0.197	0.048	0.080	0.197
**Pro_ADI Vs O_tempMIN**	**1**	F-value	0.558	0.697	0.682	0.558
	**1**	P-value	0.469	0.404	0.409	0.469

	2	F-value	0.287	0.892	0.864	0.287
	2	P-value	0.757	0.640	0.649	0.757
**O_prep Vs Pro_WSI**	1	F-value	0.640	0.800	0.780	0.640
	1	P-value	0.439	0.371	0.377	0.439
	2	F-value	0.445	1.384	1.320	0.445
	2	P-value	0.654	0.501	0.517	0.654
**Pro_WSI Vs O_prep**	1	F-value	1.313	1.641	1.557	1.313
	1	P-value	0.274	0.200	0.212	0.274
	2	F-value	1.102	3.429	3.067	1.102
	2	P-value	0.373	0.180	0.216	0.373
**O_prep Vs Pro_HUM**	1	F-value	2.138	2.673	2.460	2.138
	1	P-value	0.169	0.102	0.117	0.169
	2	F-value	2.832	8.809	6.833	2.832
	2	P-value	0.111	0.012	0.033	0.111
**Pro_HUM Vs O_prep**	1	F-value	0.000	0.000	0.000	0.000
	1	P-value	0.997	0.997	0.997	0.997
	2	F-value	0.398	1.237	1.186	0.398
	2	P-value	0.683	0.539	0.553	0.683
**O_prep Vs Pro_prep**	1	F-value	3.429	4.286	3.770	3.429
	1	P-value	0.089	0.038	0.052	0.089
	2	F-value	2.515	7.825	6.216	2.515
	2	P-value	0.136	0.020	0.045	0.136
**Pro_prep Vs O_prep**	1	F-value	0.116	0.145	0.144	0.116
	1	P-value	0.739	0.703	0.704	0.739
	2	F-value	1.579	4.913	4.211	1.579
	2	P-value	0.258	0.086	0.122	0.258
**O_prep Vs Pro_tempMIN**	1	F-value	0.023	0.029	0.029	0.023
	1	P-value	0.882	0.866	0.866	0.882
	2	F-value	0.160	0.498	0.490	0.160
	2	P-value	0.854	0.779	0.783	0.854
**Pro_tempMIN Vs O_prep**	1	F-value	2.337	2.921	2.669	2.337
	1	P-value	0.152	0.087	0.102	0.152
	2	F-value	0.551	1.713	1.616	0.551
	2	P-value	0.595	0.425	0.446	0.595
**O_prep Vs Pro_tempMEAN**	1	F-value	0.051	0.063	0.063	0.051
	1	P-value	0.826	0.801	0.802	0.826
	2	F-value	0.182	0.567	0.556	0.182
	2	P-value	0.836	0.753	0.757	0.836
**Pro_tempMEAN Vs O_prep**	1	F-value	1.499	1.874	1.766	1.499
	1	P-value	0.244	0.171	0.184	0.244
	2	F-value	0.430	1.338	1.278	0.430
	2	P-value	0.663	0.512	0.528	0.663
**O_prep Vs Pro_tempMAX**	1	F-value	0.201	0.251	0.249	0.201
	1	P-value	0.662	0.617	0.618	0.662
	2	F-value	0.605	1.882	1.766	0.605
	2	P-value	0.567	0.390	0.414	0.567

**Pro_tempMAX Vs O_prep**	**1**	F-value	0.974	1.217	1.171	0.974
	**1**	P-value	0.343	0.270	0.279	0.343
	**2**	F-value	0.589	1.832	1.722	0.589
	**2**	P-value	0.575	0.400	0.423	0.575
**O_prep Vs Pro_GSL**	**1**	F-value	0.696	0.869	0.845	0.696
	**1**	P-value	0.421	0.351	0.358	0.421
	**2**	F-value	1.067	3.319	2.979	1.067
	**2**	P-value	0.384	0.190	0.226	0.384
**Pro_GSL Vs O_prep**	**1**	F-value	2.702	3.378	3.047	2.702
	**1**	P-value	0.126	0.066	0.081	0.126
	**2**	F-value	0.804	2.502	2.302	0.804
	**2**	P-value	0.477	0.286	0.316	0.477
**O_prep Vs Pro_ADI**	**1**	F-value	1.735	2.169	2.026	1.735
	**1**	P-value	0.212	0.141	0.155	0.212
	**2**	F-value	1.377	4.284	3.738	1.377
	**2**	P-value	0.301	0.117	0.154	0.301
**Pro_ADI Vs O_prep**	**1**	F-value	0.971	1.214	1.167	0.971
	**1**	P-value	0.344	0.271	0.280	0.344
	**2**	F-value	0.628	1.955	1.830	0.628
	**2**	P-value	0.555	0.376	0.401	0.555
**Pro_WSI Vs Pro_HUM**	**1**	F-value	0.381	0.477	0.469	0.381
	**1**	P-value	0.548	0.490	0.493	0.548
	**2**	F-value	0.553	1.719	1.622	0.553
	**2**	P-value	0.594	0.423	0.444	0.594
**Pro_HUM Vs Pro_WSI**	**1**	F-value	1.522	1.902	1.791	1.522
	**1**	P-value	0.241	0.168	0.181	0.241
	**2**	F-value	1.424	4.431	3.850	1.424
	**2**	P-value	0.290	0.109	0.146	0.290
**Pro_WSI Vs Pro_prep**	**1**	F-value	0.766	0.957	0.928	0.766
	**1**	P-value	0.399	0.328	0.335	0.399
	**2**	F-value	0.436	1.355	1.293	0.436
	**2**	P-value	0.660	0.508	0.524	0.660
**Pro_prep Vs Pro_WSI**	**1**	F-value	0.417	0.521	0.512	0.417
	**1**	P-value	0.531	0.470	0.474	0.531
	**2**	F-value	0.394	1.225	1.174	0.394
	**2**	P-value	0.686	0.542	0.556	0.686
**Pro_WSI Vs Pro_tempMIN**	**1**	F-value	7.192	8.990	7.044	7.192
	**1**	P-value	0.020	0.003	0.008	0.020
	**2**	F-value	3.983	12.390	8.875	3.983
	**2**	P-value	0.058	0.002	0.012	0.058
**Pro_tempMIN Vs Pro_WSI**	**1**	F-value	2.117	2.646	2.437	2.117
	**1**	P-value	0.171	0.104	0.118	0.171
	**2**	F-value	0.132	0.412	0.406	0.132
	**2**	P-value	0.878	0.814	0.816	0.878
**Pro_WSI Vs Pro_tempMEAN**	**1**	F-value	4.757	5.946	5.009	4.757
	**1**	P-value	0.050	0.015	0.025	0.050

	2	F-value	1.529	4.756	4.094	1.529
	2	P-value	0.268	0.093	0.129	0.268
**Pro_tempMEAN Vs Pro_WSI**	1	F-value	1.619	2.024	1.898	1.619
	1	P-value	0.227	0.155	0.168	0.227
	2	F-value	0.157	0.487	0.479	0.157
	2	P-value	0.857	0.784	0.787	0.857
**Pro_WSI Vs Pro_tempMAX**	1	F-value	4.130	5.163	4.437	4.130
	1	P-value	0.065	0.023	0.035	0.065
	2	F-value	2.421	7.531	6.026	2.421
	2	P-value	0.144	0.023	0.049	0.144
**Pro_tempMAX Vs Pro_WSI**	1	F-value	0.680	0.850	0.826	0.680
	1	P-value	0.426	0.357	0.363	0.426
	2	F-value	0.334	1.041	1.004	0.334
	2	P-value	0.724	0.594	0.605	0.724
**Pro_WSI Vs Pro_GSL**	1	F-value	0.040	0.050	0.050	0.040
	1	P-value	0.845	0.824	0.824	0.845
	2	F-value	1.310	4.076	3.577	1.310
	2	P-value	0.317	0.130	0.167	0.317
**Pro_GSL Vs Pro_WSI**	1	F-value	3.598	4.497	3.933	3.598
	1	P-value	0.082	0.034	0.047	0.082
	2	F-value	8.482	26.388	14.833	8.482
	2	P-value	0.009	0.000	0.001	0.009
**Pro_WSI Vs Pro_ADI**	1	F-value	2.020	2.526	2.334	2.020
	1	P-value	0.181	0.112	0.127	0.181
	2	F-value	1.313	4.085	3.584	1.313
	2	P-value	0.316	0.130	0.167	0.316
**Pro_ADI Vs Pro_WSI**	1	F-value	0.169	0.212	0.210	0.169
	1	P-value	0.688	0.645	0.646	0.688
	2	F-value	0.120	0.373	0.368	0.120
	2	P-value	0.888	0.830	0.832	0.888
**Pro_HUM Vs Pro_prep**	1	F-value	0.042	0.052	0.052	0.042
	1	P-value	0.842	0.820	0.820	0.842
	2	F-value	0.920	2.862	2.604	0.920
	2	P-value	0.433	0.239	0.272	0.433
**Pro_prep Vs Pro_HUM**	1	F-value	1.102	1.378	1.318	1.102
	1	P-value	0.314	0.240	0.251	0.314
	2	F-value	0.465	1.447	1.377	0.465
	2	P-value	0.642	0.485	0.502	0.642
**Pro_HUM Vs Pro_tempMIN**	1	F-value	0.001	0.002	0.002	0.001
	1	P-value	0.973	0.969	0.969	0.973
	2	F-value	0.010	0.030	0.030	0.010
	2	P-value	0.990	0.985	0.985	0.990
**Pro_tempMIN Vs Pro_HUM**	1	F-value	0.124	0.155	0.154	0.124
	1	P-value	0.731	0.694	0.695	0.731
	2	F-value	1.089	3.389	3.035	1.089
	2	P-value	0.377	0.184	0.219	0.377

**Pro_HUM Vs Pro_tempMEAN**	**1**	F-value	0.073	0.092	0.092	0.073
	**1**	P-value	0.791	0.762	0.762	0.791
	**2**	F-value	0.241	0.749	0.730	0.241
	**2**	P-value	0.791	0.688	0.694	0.791
**Pro_tempMEAN Vs Pro_HUM**	**1**	F-value	0.181	0.227	0.225	0.181
	**1**	P-value	0.678	0.634	0.635	0.678
	**2**	F-value	0.691	2.149	1.999	0.691
	**2**	P-value	0.526	0.342	0.368	0.526
**Pro_HUM Vs Pro_tempMAX**	**1**	F-value	0.846	1.058	1.022	0.846
	**1**	P-value	0.376	0.304	0.312	0.376
	**2**	F-value	0.671	2.086	1.945	0.671
	**2**	P-value	0.535	0.352	0.378	0.535
**Pro_tempMAX Vs Pro_HUM**	**1**	F-value	0.291	0.364	0.360	0.291
	**1**	P-value	0.599	0.546	0.549	0.599
	**2**	F-value	0.476	1.480	1.407	0.476
	**2**	P-value	0.636	0.477	0.495	0.636
**Pro_HUM Vs Pro_GSL**	**1**	F-value	1.250	1.562	1.486	1.250
	**1**	P-value	0.285	0.211	0.223	0.285
	**2**	F-value	1.316	4.095	3.592	1.316
	**2**	P-value	0.315	0.129	0.166	0.315
**Pro_GSL Vs Pro_HUM**	**1**	F-value	2.643	3.304	2.986	2.643
	**1**	P-value	0.130	0.069	0.084	0.130
	**2**	F-value	0.658	2.049	1.912	0.658
	**2**	P-value	0.541	0.359	0.384	0.541
**Pro_HUM Vs Pro_ADI**	**1**	F-value	0.536	0.671	0.656	0.536
	**1**	P-value	0.478	0.413	0.418	0.478
	**2**	F-value	0.784	2.438	2.248	0.784
	**2**	P-value	0.486	0.295	0.325	0.486
**Pro_ADI Vs Pro_HUM**	**1**	F-value	0.002	0.003	0.003	0.002
	**1**	P-value	0.963	0.958	0.958	0.963
	**2**	F-value	0.359	1.117	1.074	0.359
	**2**	P-value	0.708	0.572	0.584	0.708
**Pro_prep Vs Pro_tempMIN**	**1**	F-value	0.758	0.947	0.918	0.758
	**1**	P-value	0.401	0.330	0.338	0.401
	**2**	F-value	1.018	3.167	2.855	1.018
	**2**	P-value	0.399	0.205	0.240	0.399
**Pro_tempMIN Vs Pro_prep**	**1**	F-value	0.000	0.000	0.000	0.000
	**1**	P-value	1.000	1.000	1.000	1.000
	**2**	F-value	2.510	7.809	6.206	2.510
	**2**	P-value	0.136	0.020	0.045	0.136
**Pro_prep Vs Pro_tempMEAN**	**1**	F-value	0.308	0.385	0.380	0.308
	**1**	P-value	0.589	0.535	0.538	0.589
	**2**	F-value	1.024	3.186	2.871	1.024
	**2**	P-value	0.397	0.203	0.238	0.397
**Pro_tempMEAN Vs Pro_prep**	**1**	F-value	0.042	0.052	0.052	0.042
	**1**	P-value	0.841	0.819	0.819	0.841

	2	F-value	2.420	7.530	6.025	2.420
	2	P-value	0.144	0.023	0.049	0.144
**Pro_prep Vs Pro_tempMAX**	1	F-value	0.670	0.837	0.815	0.670
	1	P-value	0.429	0.360	0.367	0.429
	2	F-value	0.961	2.989	2.709	0.961
	2	P-value	0.419	0.224	0.258	0.419
**Pro_tempMAX Vs Pro_prep**	1	F-value	0.165	0.206	0.204	0.165
	1	P-value	0.692	0.650	0.651	0.692
	2	F-value	2.045	6.361	5.244	2.045
	2	P-value	0.185	0.042	0.073	0.185
**Pro_prep Vs Pro_GSL**	1	F-value	0.262	0.328	0.324	0.262
	1	P-value	0.618	0.567	0.569	0.618
	2	F-value	1.583	4.926	4.221	1.583
	2	P-value	0.257	0.085	0.121	0.257
**Pro_GSL Vs Pro_prep**	1	F-value	1.674	2.092	1.958	1.674
	1	P-value	0.220	0.148	0.162	0.220
	2	F-value	0.567	1.763	1.660	0.567
	2	P-value	0.586	0.414	0.436	0.586
**Pro_prep Vs Pro_ADI**	1	F-value	5.165	6.456	5.369	5.165
	1	P-value	0.042	0.011	0.020	0.042
	2	F-value	3.082	9.589	7.304	3.082
	2	P-value	0.096	0.008	0.026	0.096
**Pro_ADI Vs Pro_prep**	1	F-value	0.079	0.099	0.099	0.079
	1	P-value	0.783	0.753	0.753	0.783
	2	F-value	0.302	0.940	0.910	0.302
	2	P-value	0.746	0.625	0.634	0.746
**Pro_tempMIN Vs Pro_tempMEAN**	1	F-value	0.093	0.116	0.116	0.093
	1	P-value	0.766	0.733	0.734	0.766
	2	F-value	1.242	3.863	3.412	1.242
	2	P-value	0.334	0.145	0.182	0.334
**Pro_tempMEAN Vs Pro_tempMIN**	1	F-value	0.160	0.201	0.199	0.160
	1	P-value	0.696	0.654	0.655	0.696
	2	F-value	2.704	8.412	6.588	2.704
	2	P-value	0.120	0.015	0.037	0.120
**Pro_tempMIN Vs Pro_tempMAX**	1	F-value	0.161	0.201	0.199	0.161
	1	P-value	0.696	0.654	0.655	0.696
	2	F-value	1.655	5.148	4.384	1.655
	2	P-value	0.244	0.076	0.112	0.244
**Pro_tempMAX Vs Pro_tempMIN**	1	F-value	0.319	0.399	0.393	0.319
	1	P-value	0.583	0.528	0.530	0.583
	2	F-value	1.675	5.212	4.431	1.675
	2	P-value	0.241	0.074	0.109	0.241
**Pro_tempMIN Vs Pro_GSL**	1	F-value	0.748	0.935	0.907	0.748
	1	P-value	0.404	0.334	0.341	0.404
	2	F-value	0.020	0.061	0.061	0.020
	2	P-value	0.980	0.970	0.970	0.980

**Pro_GSL Vs Pro_tempMIN**	**1**	F-value	1.030	1.288	1.235	1.030
	**1**	P-value	0.330	0.256	0.266	0.330
	**2**	F-value	0.529	1.646	1.557	0.529
	**2**	P-value	0.606	0.439	0.459	0.606
**Pro_tempMIN Vs Pro_ADI**	**1**	F-value	0.000	0.000	0.000	0.000
	**1**	P-value	0.999	0.999	0.999	0.999
	**2**	F-value	1.287	4.004	3.521	1.287
	**2**	P-value	0.322	0.135	0.172	0.322
**Pro_ADI Vs Pro_tempMIN**	**1**	F-value	1.628	2.035	1.908	1.628
	**1**	P-value	0.226	0.154	0.167	0.226
	**2**	F-value	0.916	2.849	2.593	0.916
	**2**	P-value	0.435	0.241	0.273	0.435
**Pro_tempMEAN Vs Pro_tempMAX**	**1**	F-value	0.622	0.777	0.758	0.622
	**1**	P-value	0.446	0.378	0.384	0.446
	**2**	F-value	1.918	5.967	4.970	1.918
	**2**	P-value	0.202	0.051	0.083	0.202
**Pro_tempMAX Vs Pro_tempMEAN**	**1**	F-value	1.080	1.350	1.292	1.080
	**1**	P-value	0.319	0.245	0.256	0.319
	**2**	F-value	1.473	4.583	3.964	1.473
	**2**	P-value	0.280	0.101	0.138	0.280
**Pro_tempMEAN Vs Pro_GSL**	**1**	F-value	0.316	0.395	0.389	0.316
	**1**	P-value	0.585	0.530	0.533	0.585
	**2**	F-value	0.343	1.067	1.028	0.343
	**2**	P-value	0.719	0.587	0.598	0.719
**Pro_GSL Vs Pro_tempMEAN**	**1**	F-value	0.236	0.295	0.293	0.236
	**1**	P-value	0.636	0.587	0.589	0.636
	**2**	F-value	0.210	0.652	0.637	0.210
	**2**	P-value	0.815	0.722	0.727	0.815
**Pro_tempMEAN Vs Pro_ADI**	**1**	F-value	0.003	0.004	0.004	0.003
	**1**	P-value	0.955	0.949	0.949	0.955
	**2**	F-value	0.728	2.266	2.100	0.728
	**2**	P-value	0.509	0.322	0.350	0.509
**Pro_ADI Vs Pro_tempMEAN**	**1**	F-value	1.601	2.002	1.879	1.601
	**1**	P-value	0.230	0.157	0.170	0.230
	**2**	F-value	1.739	5.409	4.574	1.739
	**2**	P-value	0.230	0.067	0.102	0.230
**Pro_tempMAX Vs Pro_GSL**	**1**	F-value	0.025	0.031	0.031	0.025
	**1**	P-value	0.877	0.860	0.860	0.877
	**2**	F-value	0.371	1.155	1.110	0.371
	**2**	P-value	0.700	0.561	0.574	0.700
**Pro_GSL Vs Pro_tempMAX**	**1**	F-value	0.011	0.014	0.014	0.011
	**1**	P-value	0.917	0.905	0.905	0.917
	**2**	F-value	0.156	0.485	0.477	0.156
	**2**	P-value	0.858	0.785	0.788	0.858
**Pro_tempMAX Vs Pro_ADI**	**1**	F-value	0.070	0.088	0.087	0.070
	**1**	P-value	0.796	0.767	0.768	0.796

	**2**	F-value	0.242	0.752	0.733	0.242
	**2**	P-value	0.790	0.687	0.693	0.790
**Pro_ADI Vs Pro_tempMAX**	**1**	F-value	2.817	3.521	3.163	2.817
	**1**	P-value	0.119	0.061	0.075	0.119
	**2**	F-value	2.233	6.948	5.642	2.233
	**2**	P-value	0.163	0.031	0.060	0.163
**Pro_GSL Vs Pro_ADI**	**1**	F-value	8.374	10.467	7.940	8.374
	**1**	P-value	0.013	0.001	0.005	0.013
	**2**	F-value	3.441	10.705	7.951	3.441
	**2**	P-value	0.078	0.005	0.019	0.078
**Pro_ADI Vs Pro_GSL**	**1**	F-value	0.388	0.485	0.477	0.388
	**1**	P-value	0.545	0.486	0.490	0.545
	**2**	F-value	0.310	0.964	0.932	0.310
	**2**	P-value	0.741	0.618	0.628	0.741

Key: Rows highlighted in grey indicate significant p-values ($p \leq 0.05$). Statistical probability values (i.e., p-values) are based on the Augmented Dickey–Fuller (ADF) test.

Supplementary Table 3: OPLS model fit summary parameters

YA

Component	R2X	R2X(cum)	R2Y	R2Y(cum)	Q2	Limit	Q2(cum)	Sig.
0	Cent.							
1	0.33	0.33	0.566	0.566	0.286	0.05	0.286	R1
2	0.199	0.529	0.198	0.764	0.231	0.05	0.451	R1

YI

Component	R2X	R2X(cum)	R2Y	R2Y(cum)	Q2	Limit	Q2(cum)	Sig.
0	Cent.							
1	0.131	0.131	0.529	0.529	-0.189	0.05	-0.1	NS
2	0.226	0.357	0.111	0.641	-0.267	0.05	-0.21	NS

YM

Component	R2X	R2X(cum)	R2Y	R2Y(cum)	Q2	Limit	Q2(cum)	Sig.
0	Cent.							
1	0.358	0.358	0.336	0.336	0.0301	0.05	0.0301	NS
2	0.127	0.485	0.197	0.533	-0.247	0.05	-0.0668	NS

Supplementary Table 4: Kendall W test of concordance and Friedman nonparametric hypothesis test

**Ranks**	
	Mean Rank
SVM	2.67
XGB	2.08
RF	2.00
OPLS	3.25

**Kendall's W Test Statistics**	
N	12
Kendall's W[a]	0.203
Chi-Square	7.300
df	3
Asymp. Sig.	0.063

**Friedman Test Statistics[b]**	
N	12
Chi-Square	7.300
df	3
Asymp. Sig.	0.063

a. Kendall's Coefficient of Concordance
b. Friedman Test
